# Population co-divergence in common cuttlefish (*Sepia officinalis*) and its dicyemid parasite in the Mediterranean Sea

**DOI:** 10.1038/s41598-019-50555-9

**Published:** 2019-10-04

**Authors:** Marie Drábková, Nikola Jachníková, Tomáš Tyml, Hana Sehadová, Oleg Ditrich, Eva Myšková, Václav Hypša, Jan Štefka

**Affiliations:** 1 Faculty of Science, University of South Bohemia, České Budějovice, Czech Republic; 2 Biology Centre, CAS, v.v.i., České Budějovice, Czech Republic; 3 Faculty of Science, Masaryk University, Brno, Czech Republic

**Keywords:** Biogeography, Coevolution, Evolutionary biology

## Abstract

Population structure and biogeography of marine organisms are formed by different drivers than in terrestrial organisms. Yet, very little information is available even for common marine organisms and even less for their associated parasites. Here we report the first analysis of population structure of both a cephalopod host (*Sepia officinalis*) and its dicyemid parasite, based on a homologous molecular marker (cytochrome oxidase I). We show that the population of common cuttlefish in the Mediterranean area is fragmented into subpopulations, with some areas featuring restricted level of gene flow. Amongst the studied areas, Sardinia was genetically the most diverse and Cyprus the most isolated. At a larger scale, across the Mediterranean, the population structure of the parasite shows co-diversification pattern with its host, but a slower rate of diversification. Differences between the two counterparts are more obvious at a finer scale, where parasite populations show increased level of fragmentation and lower local diversities. This discrepancy can be caused by local extinctions and replacements taking place more frequently in the dicyemid populations, due to their parasitic lifestyle.

## Introduction

In marine organisms, genetic structure is usually supposed to be determined by various extrinsic factors unique to this environment. Among the most typical factors are the lack (or rare occurrence) of obvious dispersal barriers or boundaries compared to terrestrial systems^1^. The lack of dispersal barriers should, in theory, lead to the maintenance of large effective populations sizes, spanning vast areas of suitable habitats and showing low level of inter-population genetic variation^2^. However, while empirical data confirmed this view for some organisms (i.e., *Architeuthis dux*^3^, *Homarus gammarus*^4^, *Thunnus alalunga*^5^), others showed surprisingly high diversification on a smaller scale than would be expected (i.e., Dinoflagellate *Alexandrium minutum*^6^, *Sepia esculenta*^7^, for more examples see ref.^1^). Palumbi *et al*.^1^ listed several factors possibly responsible for such diversification. They include biological traits as well as physical barriers (mainly ocean/sea currents). Since then, many studies were carried out on a broad taxonomic range of marine organisms in different oceans and seas, revealing a high variety of reconstructed genetic patterns and their relationships to the oceanographic conditions, showing that genetically homogeneous populations are not the only option in marine organisms^8^.

Mediterranean Sea, with its extremely rich biodiversity and a long history of research interest, belongs among the best mapped marine regions. As a consequence, the oceanographic processes (currents and discontinuities) are well known^9,10^ and their possible influence on population genetic connectivity has been investigated for many organisms (e.g. reviews focused on fish and benthic invertebrates^11,12^). Mediterranean Sea has been traditionally divided into Western and Eastern Basins (here shortened as WB and EB), connected by the Strait of Sicily and possibly also by the Strait of Messina. Water currents in the WB are defined by two major oceanographic fronts, the Almeria-Oran front (AO front) near Gibraltar and the North Balearic front (NB front) near Balearic islands. In the EB, the two major barriers are represented by the Otranto Strait, separating Adriatic Sea, and the Greek islands, forming the Aegean front^9,10^. In a recent meta-analysis Pascual *et al*.^12^ showed that the relationship between population structure and oceanographic features in Mediterranean Sea varies considerably across different species and is largely determined by the life history of the given organism. Particularly, the presence/absence of a pelagic larva, and duration of this phase, is an important factor in the dispersal capacity and therefore determines population structure. For example, the organisms with low dispersal capabilities show significant genetic differentiation, but their population structure is not determined by the oceanographic fronts^12^. High variety of the reconstructed genetic patterns for different organisms shows that understanding genetic differentiation and gene flow in marine conditions will require number of genetic studies on a rich variety of biologically different organisms.

Considering strong dependence of parasites on their hosts, and obvious role of the host in parasites’ dispersal, the factors driving population diversity of free-living marine organisms should, in turn, affect the genetic diversity and structure of their parasites. However, the degree of such interdependency is yet unclear. While in the terrestrial systems, comparative studies on both host and parasite population structures are more common (e.g.^13,14^), marine surveys are limited to a few studies, usually involving complex multi-host systems^15^, or parasites of sessile hosts, lacking opportunities for co-dispersal^16^. The studies performed so far indicate that the answer to this question is likely to be dependent on the particular model and its biological traits. For example, Blasco-Costa and Poulin^17^, reviewing studies on 16 trematode species, concluded that the host mobility is the main determinant of the parasite genetic diversification. A slightly different view was presented by Maze-Guilmo *et al*.^18^. Based on their meta-analysis of a broader spectrum of parasites, they demonstrated that the outcome of such parasite-host comparison is dependent on various biological traits (i.e. reproduction mode, presence/absence of a larval stage). For example, they suggest that, generally, parasites tend to show lower genetic differentiation than the host, particularly in hermaphroditic parasites with asexual reproduction phase, whereas for gonochoristic groups the genetic differentiation is often the same or even higher than in the host. Maze-Guilmo *et al*.^18^ explain this discrepancy by a lower number of dispersal events required for successful host colonization by hermaphroditic parasites.

Analysing the impact of host population structure in multi-host systems, such as in digenean parasites, is complicated by the presence of intricate networks often including migratory or terrestrial hosts^19^. On the contrary, comparative studies of single-host-parasite associations provide a more straightforward approach, with the capacity to address such questions as: Is the overall population structure of a parasite mirroring that of its host due to their shared dispersal? May the structure of parasite’s local subpopulation differ from the host due to local extinctions and reinfections? Would such extinctions result in a decrease of genetic diversities within local populations of the parasites? Here, we address these questions using a model of host-parasite pair, Common cuttlefish (*Sepia officinalis*) and its parasites from a rarely studied group Dicyemida. This model allows for addressing the general issues of genetic diversification in marine environment but also specifically the relationship between diversities of the host and parasite. To our best knowledge, this study introduces the first single-host model entirely bound to the marine environment and involving a free-living host.

Common cuttlefish (*S*. *officinalis*) is an important species for fisheries in the Mediterranean Sea and the neighbouring Atlantic coast, which makes it one of the few marine organisms for which considerable amount of data is available. Considering the span and connectivity of the suitable habitats, together with the mobility of adults, cuttlefish could in theory maintain large continuous population, with isolation by distance as a main pattern of the genetic structure. However, the known biological features of the cuttlefish suggest that more complex population structure, affected by other factors than mere geographic distance, might be expected. Cuttlefish is a benthic cephalopod, that, unlike most octopuses and squids, does not possess an obvious pelagic early phase of life (e.g. larvae) as a mean of dispersal. Moreover, its lifespan of only one to two years suggests a quick turnover of the residing population. These life history traits may predispose cuttlefish to formation of fragmented populations (e.g. a network of relatively isolated subpopulations with poor genetic exchange). The few studies carried out for this species in general support this view, indicating substantial level of structuring across the whole Mediterranean Sea and the effect of Isolation By Distance (IBD). However, they do not provide any conclusive view, since based on the used molecular marker, they provide slightly different pictures. The first indication of significant subpopulation structuring in this organism came from the allozyme analysis carried out by Perez-Losada *et al*.^20^, which revealed clinal changes of the analyzed allozymes between Mediterranean and Atlantic localities but did not indicate any clear subdivision associated with any oceanic front in the Mediterranean. A significant increase in the genetic distances across the AO front was indicated by the following microsatellite study, which, however, covered only the coasts of the Iberian peninsula^21^. The third study, using mitochondrial gene for COI, brought the most complex analysis covering a large area from Greece to the Atlantic coast of Portugal. It confirmed the general picture of a highly fragmented population with restricted gene flow and possible effect of some oceanographic factors^22^.

Even less information is available on intraspecific genetic diversity of the organisms associated with the cuttlefish as a host. Perhaps the closest topic addressed for the Mediterranean cephalopods is the study of symbiotic *Vibrio* in sepiolid squids, showing independence of the host and symbiont genetic structures^23^. In dicyemids, their life history parameters indicate much higher degree of host-dependence. Dicyemids are endogenous parasites only found in the renal organs of benthic cephalopods, such as cuttlefish or octopus, and they are completely dependent on their host for growth and reproduction^24^. Both asexual and sexual reproduction take place inside the host, whereas only a short-lived larva is expected to serve as a means of dispersal within the host population^25^. This dependency of dicyemids on their host indicates that their inter-host transfer can only occur on short distances and their population structure will thus be strongly determined by the host. More specifically, we expect that their dispersion across long distances is driven entirely by the host and the gross genetic structure of the dicyemids will thus correspond to that of the cuttlefish. However, since they are spreading between hosts by environment (possibly by a free swiming larva, that hovers near sea floor) and do not transmit vertically (see^26^) their population structure could deviate from the host’s pattern within local populations. Almost no information is currently available on dicyemid population structure or genetic diversity to test these questions. Up to now, a little more than 120 species of dicyemids have been described^27^, whereas only a few 18S rDNA sequences from dicyemids associated with eastern Pacific cephalopods^28^ and from *Dicyemennea eledones* associated with *Eledone cirrhosa* in the Mediterranean^29^ have been published. Despite the fact, that dicyemids were also suggested as suitable tags in phylogeographic parasitological research^30,31^, only several sequences usable for population studies are publicly available in GenBank, but has not been published (such as the sequences of dicyemids from Australian cephalopods; Catalano 2013 unpublished).

## Materials and Methods

**Figure 1:**
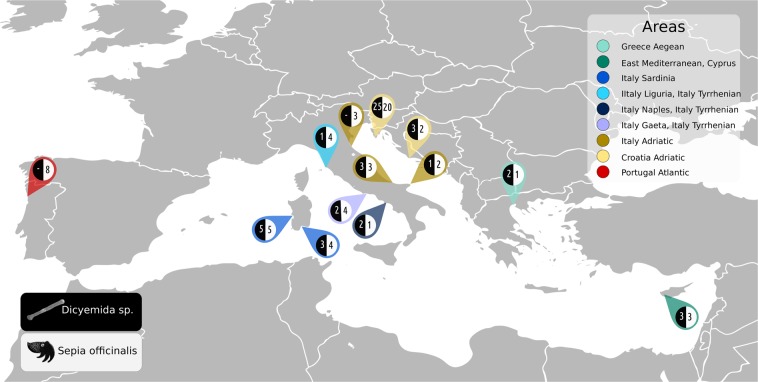
Map of sampling area showing the number of host specimens and parasite samples from each locality. Dicyemids white font, black background, their hosts common cuttlefish (*Sepia officinalis*) black font white background. The colours of the pins correspond to population network in Fig. 2.

Samples were collected at 13 localities in the in the Mediterranean area and the Atlantic coast of Portugal (Fig. 1) during multiple sampling trips from 2014 to 2017. Complete list of the samples and localities is available in Supplementary Table S1. Freshly killed cephalopods were purchased on local fish markets and geographic origin of the samples was confirmed with the retailers to rule out mixing samples from the adjacent localities. The purchased samples were kept on ice until dissection. A piece of arm muscle tissue was excised and stored in pure ethanol as a reference for the host. Mantle was opened from the ventral side and renal tissue was carefully transferred into a dish or a falcon tube containing artificial seawater (prepared according to^24^) to dislodge attached dicyemids. Liquid with the dislodged dicyemids was transferred into a micro-tube and centrifuged at low speed to concentrate dicyemids in the solution. Supernatant was removed, additional amount of liquid with dicyemids was added and the centrifugation was repeated. Finally, supernatant was removed and 1 ml of pure ethanol was added for storage.

Slides for morphological examination were prepared according to the protocol of Pascual *et al*.^32^. In summary, a piece of renal tissue was smeared on a coverslip and fixed in Boiun’s fluid for up to 20 minutes, then stored in 70% ethanol until staining with hematoxylin-eosin and mounting in Canada balsam.

One sample with live dicyemids in good condition (SOIN1) was fixed and stained for confocal microscopy to provide illustrative photos of dicyemids from *Sepia officinalis*.

Prior to DNA extraction, samples were removed from ethanol and let to dry. For the hosts, DNA extractions from individual specimens were performed by DNeasy Blood and tissue kit (QIAGEN). For the parasites, pooled samples (each obtained from an individual host, but containing multiple individual dicyemids) were extracted by QIAamp DNA Micro Kit (QIAGEN). In both cases, the protocol provided by the manufacturer was followed. For cephalopod hosts, universal primers F1490^33^ and H7005^34^ were used for amplification of a 1100 bp fragment of the mitochondrial cytochrome oxidase I (COI) gene. For dicyemids, new specific primers were designed based on COI gene sequence extracted from a preliminary draft assembly of a cuttlefish renal tissue transcriptome (see Supplementary Methods for details). To supplement COI based species delimitation of the studied dicyemid parasite with a nuclear locus, we amplified and sequenced 1330 bp fragment of 18S rDNA in 23 samples across our population sets with primers F3 and R2 published in^28^. Standard PCR protocol with Taq polymerase was followed (for PCR conditions and primer sequences see Supplementary Table S2). PCR products were visualised on 1% agarose gel, enzymatically cleaned (by 2 μl FastAP and 0.5 μl ExoI enzymes with 2.5 μl H_2_O per reaction added and incubated for 15 minutes at 37 °C in thermocycler) and sequenced with PCR primers on the ABI analyzer (Thermo Fisher). Each sample was sequenced in both directions. Where necessary (in the case of the cephalopod host), specific sequencing primers were used to obtain the full length of the sequence (specifically designed primers for sequencing cephCOI11F, cephCOI14R, for primer sequences see Supplementary Table S2).

The sequences were assembled and trimmed in Geneious v. 11.1 (https://www.geneious.com/). Mafft v. 7^35^ was used for translation guided alignment of sequences. To evaluate *Sepia* diversity in a broader context, publicly available sequences were added to the sequences obtained in this study (Accession numbers in Supplementary Table S3) and phylogenetic trees were computed with IQ-TREE web server^36^ with auto model selection^37^. Ultrafast boostrap and aLRT statistics were computed as node supports^38^.

To visualize the geographic diversity of populations, haplotype networks were designed in PopArt software^39^ using Median Joining Network algorithm^40^. One reference sequence (AB2401555) of *Sepia officinalis* covering the whole length of the alignment was added to the sequences obtained in this study to verify correct assignment of all samples to *S. officinalis* species in the population network. To show *S. officinalis* population structure across its dispersal area, publicly available sequences were added to the alignment, which was however shorter (473 bp) due to only partial overlap between the sequences (Accession numbers in the Supplementary Table S3). As an alternative to haplotype networks, the population structure information of the host and parasite was complemented using a Principal Coordinates Analysis (PCoA, implemented as cmdscale in R package stats) and AMOVA with significance calculated by a permutation test (calculated in R package poppr^41^). Portuguese populations were omitted from AMOVA analyses due to the lack of parasite data.

To characterize the diversity of populations, summary statistics (Haplotype diversity, Nucleotide diversity, Theta, Dxy^42^), neutrality tests (Tajima’s D^43^), Fu and Li’s D* and F*^44^ and Fu’s Fs^45^) and several population size change statistics (Raggedness^46^), Ramos-Onsins test^47^ were obtained in DNASP ver. 5^48^. Pairwise Fst values and their significance was obtained by 10000 permutations in Arlequin^49^. Due to the fact that population sample sizes vere highly unequal in some cases (see Table 1), we also calculated these statistics for populations randomly downsized to a maximum of 5 sequences per population.

**Table 1:** Results of population statistic approaches: basic population metrics, population size changes and dynamics.

general overview		all sampled populations		Portugal	Sardinia		Italy Tyrrhenian		Italy Adriatic		Croatia Adriatic		East Mediterranean Cyprus		Greece Aegean	
		host	parasite	host	host	parasite	host	parasite	host	parasite	host	parasite	host	parasite	host	parasite
number of sequences		60	50	8	9	8	9	5	8	4	22	28	3	3	1	2
positions		864	777	864	864	777	864	777	864	777	864	777	864	777	864	777
polymorphic (segregating) sites	S	61	41	11	32	19	8	6	8	9	15	6	1	1	—	8
number of haplotypes	h	32	20	7	7	4	4	3	7	2	12	7	2	2	1	2
haplotype diversity	Hd	0,963	0,860	0,964	0,944	0,821	0,694	0,800	0,964	0,500	0,926	0,577	0,667	0,667	—	1
nucleotide diversity	Pi	0,013	0,008	0,004	0,014	0,013	0,004	0,004	0,004	0,006	0,005	0,001	0,001	0,001	—	0,010
Theta per sequence (from S)	Theta-W	13,1	9,2	4,2	11,8	7,3	2,9	2,9	3,1	4,9	4,1	1,5	0,7	0,7	—	8
average pairwise differences	k	11,4	6	3,2	12,3	9,8	3,1	3,4	3,2	4,5	4	0,7	0,7	0,7	—	8
Raggedness	r	0,01	0,03	0,06	0,077	0,208	0,169	0,28	0,086	—	0,024	0,154	—	—	—	—
Ramos-Onsins and Rozas	R2	0,089	0,07	0,115	0,168	0,257	0,181	0,259	0,166	—	0,119	0,067	—	—	—	—
Fu's	Fs	−6,404	−3,568	−*3,05*	0,719	4,117	1,337	1,569	−*3,05*	—	−3,191	−*4,386*	—	—	—	—
Fu and Li	D*	−1,411	−0,289	−1,299	−0,092	*1,582*	1	1,241	0,219	—	−0,804	−1,832	—	—	—	—
Fu and Li	F*	−1,286	−0,743	−1,417	−0,013	*1,795*	0,895	1,286	0,238	—	−0,775	−2,061	—	—	—	—
Tajima's D	D	−0,526	−1,213	−1,21	0,239	1,716	0,173	1,241	0,202	—	−0,336	−1,628	—	—	—	—

values significant at P < 0.02 in italics.

*r. R2, Fu and Li test, Fu’s Fs and Tajima’s D statistics were run for populations with at least 5 sequencess.

To test for correlation between population divergence of the host and its parasite, we estimated a linear regression of their pairwise population genetic distances (average number of nucleotides and Nei’s Da^42^) in R software^50^. As in AMOVA, Portuguese population was not included in the correlations due to the lack of parasite data. Due to the extremely small sample size Greek population was excluded from the regression analysis. Mantel test implemented in adegenet R package^51^ was used to test for Isolation By Distance (IBD) between individuals, separately for parasite and host (again excluding Portuguese population). This test compared the similarity of matrices of genetic (Gst^52^) and geographic (marine distance in km, i.e. shortest straight sea route along the coast between localities measured manually on a map) distances for individual samples (R code available in the Supplementary Methods). To control for the effect of distant populations with small sample size on the results of Mantel test, we re-calculated the test on a dataset excluding Greek and Cyprus populations.

### Data accessibility

COI haplotypes newly acquired in this study of both host and parasite and 18S (small ribosomal subunit) sequence of parasite are stored in GenBank (Accession numbers: dicyemid 18S MN066345-MN0666367, dicyemid COI MN069252-MN069301 and MN310702-MN310704, cuttlefish host COI MN069190-MN069251). Overview of all sequences used in this study with corresponding accession numbers (both from public database and produced in this study) are also provided in Supplementary Table S3.

## Results

In this study, we successfully sequenced and examined 60 individuals of the cephalopod host and 50 samples of their dicyemid parasites.

Two cuttlefish specimens from Sicily (Accession numbers MN069250 and MN069251) were found to be genetically distinct from the rest of the *S*. *officinalis* specimens in this study and from available *S*. *officinalis* sequences in public databases (approximate distance of 10% compared to other *S*. *officinalis* samples based on a phylogenetic analysis, Supplementary Fig. S1). The Sicilian samples possibly represent a different *Sepia* species or subspecies and were therefore not included in the subsequent population analyses.

**Figure 2:**
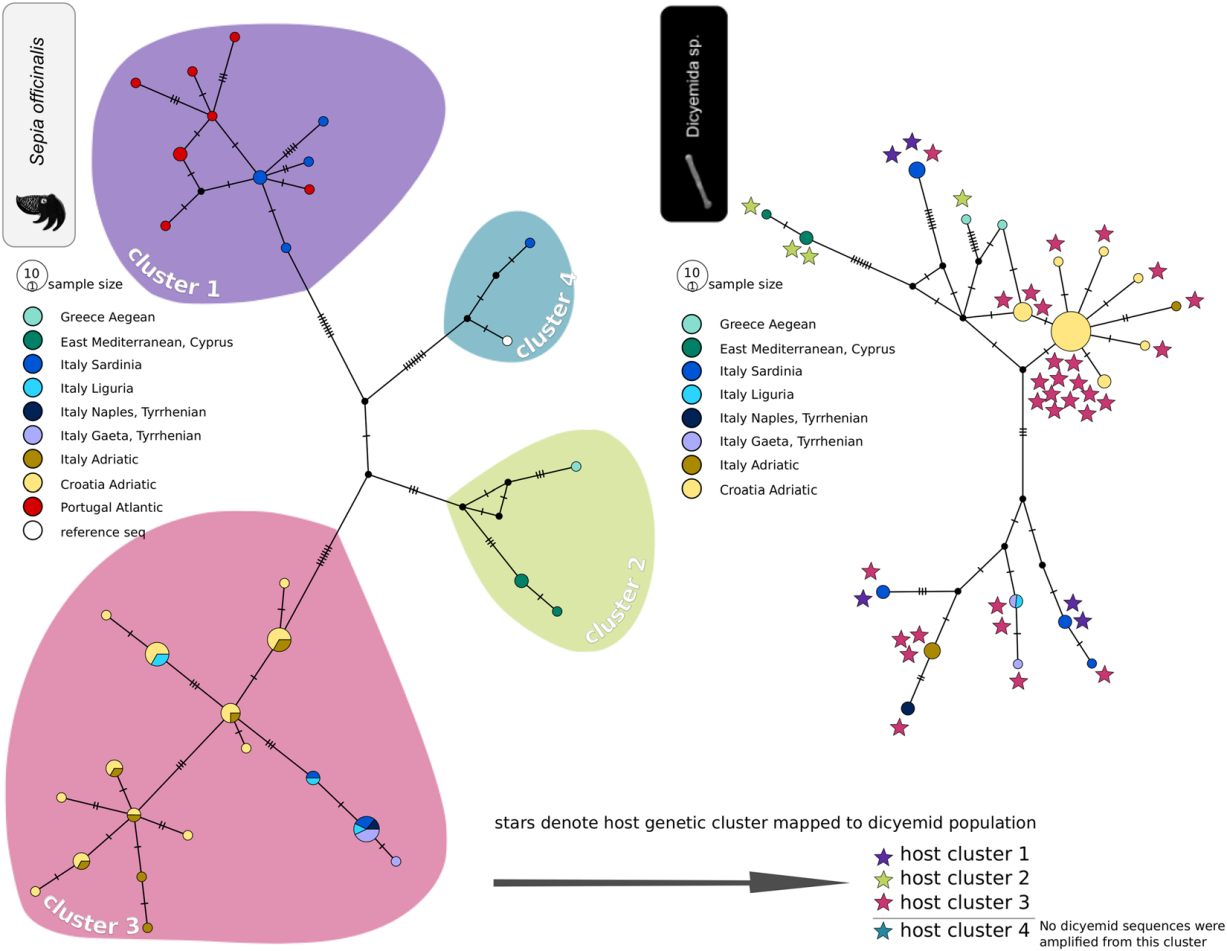
Population net (PopART, median joining network) of the host, *Sepia officinalis* (on the left), and its associated dicyemids (on the right). Stars denote the host cluster that dicyemids were associated with.

**Figure 3:**
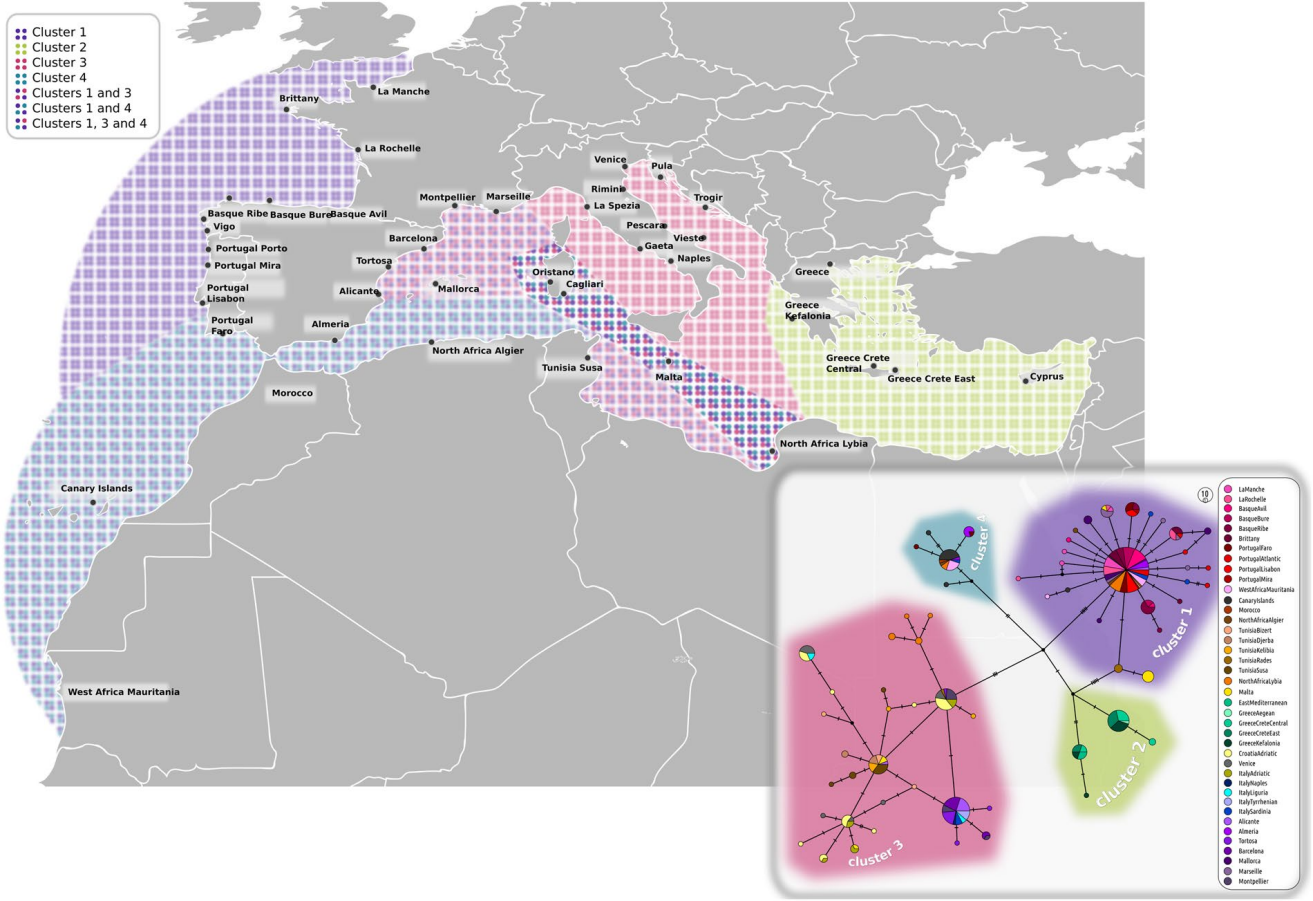
*Sepia officinalis* structure throughout its distribution. Colored areas correspond to genetic clusters shown on population network.

Haplotypes of common cuttlefish in the studied area form a fragmented structure with many missing haplotypes and most haplotypes are represented by one or a few individuals (Fig. 2), indicating high overall genetic diversity within the sampled area. At the most general level, the population structure is comprised of four main distinct clusters seen both in the haplotype network (Fig. 2) and in the PCoA (Supplementary Fig. S2). Two of the clusters encompass the majority of the obtained samples; one is found mostly in Sardinia and at the Atlantic coast of Portugal, and the other is found in the Adriatic and Tyrrhenian Seas. Two additional isolated groups are represented by only a few samples. One is represented by a single specimen found in Sardinia and is most similar to the selected *Sepia officinalis* reference sequence (see the methods), the other includes a few samples from the Eastern Mediterranean in Cyprus and Aegean sea in Greece (median joining network, Fig. 2 left). Sardinia shows surprisingly diverged set of haplotypes (Table 1) belonging to several COI clusters (Supplementary Fig. S3). In contrast, the Adriatic Sea population, is characterized by higher number but lower nucleotide diversity of their haplotypes (Table 1). Correspondingly, all four neutrality tests produced negative values (albeit statistically non-significant) for the Croatian population and significantly negative value of the Fu’s Fs in the Italian Adriatic population. Although the Fst values expressing connectivity to the closest mainland shore, may potentially be biased by low sample numbers, they point into the same direction as the nucleotide diversities (Fst 0.409 for Sardinia vs. Tyrrhenian Sea and 0.031 for Adriatic Sea between Italian and Croatian coasts; Table 2). When the dataset is extended with the *S. officinalis* sequences across the distribution range available in the GenBank, the population network consists of the same four clusters as seen in our data (Fig. 3). In the extended dataset, Adriatic and Atlantic clusters mix in the WB, one cluster is distributed only in the East Mediterranean (Cyprus, Greece) and one cluster, originally represented by only one sample found on Sardinia, is now also found on the North African coast together with Atlantic cluster.

**Table 2:** Population pairwise comparison for host (upper right triangle) and its parasite (lower right triangle) by Fst and Dxy.

	Croatia Adriatic (22)	Sardinia (9)	Italy Tyrrhenian (9)	Portugal (8)	Italy Adriatic (8)	Cyprus (3)
**Fst**						
Croatia Adriatic (28)		*0.546*	*0.352*	*0.826*	0.031	*0.785*
Sardinia (8)	*0.599*		*0.409*	*0.238*	*0.476*	*0.493*
Italy Tyrrhenian (5)	*0.853*	*0.251*		*0.839*	*0.45*	*0.861*
Portugal -	—	—	—		*0.849*	*0.86*
Italy Adriatic (4)	*0.788*	0.162	0.121	—		*0.844*
Cyprus (3)	*0.935*	*0.469*	*0.822*	—	*0.777*	
**Dxy**						
Croatia Adriatic (28)		0.018	0.006	0.025	0.004	0.019
Sardinia (8)	0.011		0.015	0.012	0.018	0.020
Italy Tyrrhenian (5)	0.010	0.012		0.022	0.007	0.021
Portugal -	—	—	—		0.025	0.021
Italy Adriatic (4)	0.008	0.012	0.006	—		0.019
Cyprus (3)	0.019	0.017	0.018	—	0.017	

Italy Tyrrhenian contains localities of Liguria, Gaeta and Naples. Greek samples are not included because of low sample size. Significant values of Fst in italics at level p = 0.05.

**Figure 4:**
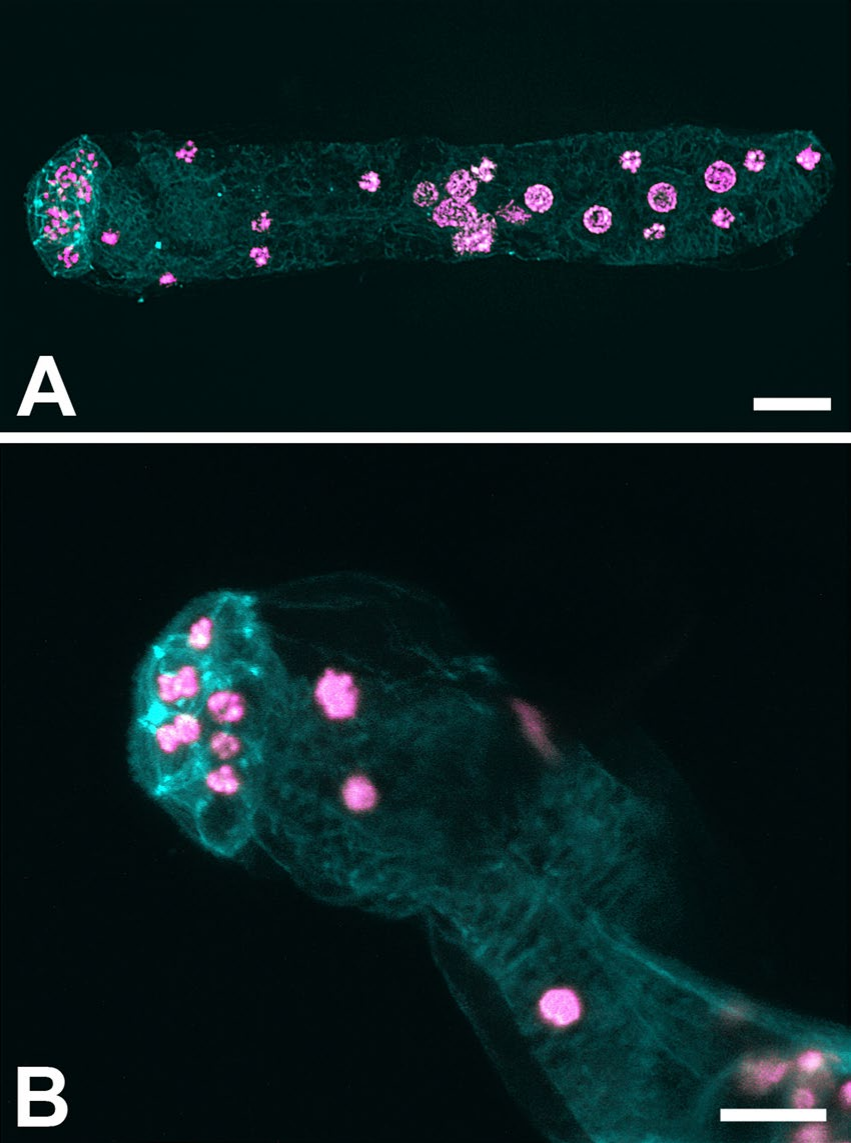
Dicyemid from *Sepia officinalis*. Confocal microscope images (DAPI/pink and phalloidin/cyan staining. (**a**) whole animal, (**b**) close up of head (calotte) with visible nuclei. The scale bar represents 10 µm.

Based on morphological examination of the stained preparations, 18S sequence and confocal microscopy (Fig. 4), we tentatively determined the species of COI sequenced dicyemids as *Pseudicyema truncatum* Whitman, 1883. Despite there is a possibility of mixed infections in the host renal organ^25^, the specificity of the designed COI primer pair seems to be very high and seems to amplify mostly one prevalent species of dicyemid. Only in 3 cases (samples SOIC4, SOIR11 and SOIR13; not included in the population dataset) the resulting sequences seemed to belong to a different dicyemid species, possibly *Dicyema* sp. or *Dicyemennea* sp. (Accession numbers MN310702-MN310704). In three cases (SOCT1, SOIC3, SOKL1) the resulting sequencing chromatogram showed double peaks in one position (a different position in each sequence) which suggests that more than one COI haplotype may be sometimes present in the sample, possibly because multiple individuals were used for DNA extraction. We arbitrarily chose the higher peak for nucleotide assignment for further analyses. We verified the species identity of our COI sequenced samples with 18S sequencing. All 18S sequences, except one (sample originating in Italia, Adriatic coast, Rimini; SOIR11), were identical to each other and were also identical to one of the *Pseudicyema truncatum* sequences available in genbank (LT669919, 1017 bp, Souidenne *et al*. 2017 unpublished). However, there is only 97% match to the other available *Pseudicyema truncatum* sequence (LT669870, 1175 bp, Souidenne *et al*. 2017 unpublished). SOIR11 sample showed only 94% match to other acquired 18S sequences and was not closely related to any available dicyemid 18S sequence, supporting the view that this cuttlefish specimen was infected by a different dicyemid species to the one this study focused on. A list of samples for which 18S sequence is available is provided in Supplementary Table S1. It is worth noting, that the number of reliably described sequences available for dicyemid species in public databases is very limited and most of them originate from Australian, Japanese and North Pacific waters making the comparisons to the Mediterranean of limited use.

Population structure of the parasite showed an interesting difference in comparison to the host. In majority of the comparisons, the basic population characteristics (Hd, Pi statistics, Table 1) suggested lower level of the local diversities in the parasite subpopulations when compared to the host (whole dataset, Sardinia and Croatian Adriatic). In the haplotype network, this reduced diversity is reflected by more compact clustering, particularly well expressed by the central haplotype from the Adriatic Sea, which represents 18 samples.

Dicyemid population structure was also partitioned into several clusters (haplotype network in Fig. 2 right, PCoA plot in Supplementary Fig. S2). Although only some of the clusters showed geographical specificity, high level of haplotype sorting between sampling sites was seen (Fig. 2 right). With the exception of two Italian localities (Liguria and Tyrrhenian), no localities shared COI haplotypes. Haplotypes from East Mediterranean (Cyprus) were separated by several mutations, similarly to the host dataset. Largest diversity was seen in the samples from Sardinia with haplotypes scattered in several parts of the network. Unfortunately, no parasite data were available from Portugal to compare with the host. Samples from Croatian Adriatic formed a compact star-like cluster suggesting possible recent expansion. Unlike the host population, Fst value between the two Adriatic coasts (Croatia and Italy) was high (Fst = 0.788, Table 2). This is in agreement with the lack of haplotype sharing between the coasts. Calculation using downsampled population sizes (N = 5) produced similar values of pairwise Fst distances (Supplementary Table S4).

Neutrality tests for parasites showed significant values in two cases. On Sardinia two tests (Fu an Li’s D and F) revealed lack of low and high frequency polymorphisms, which is usually interpreted either as a sign of past population bottlenecks or population admixture^53^. Similarly to the host, all neutrality test statistics showed negative values in the Croatian Adriatic, with the Fu’s Fs producing statistically significant value (Table 1).

**Figure 5:**
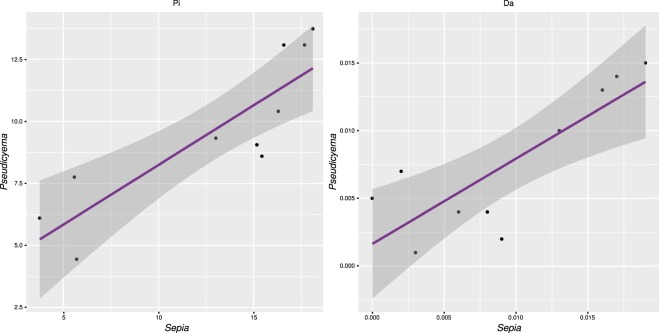
Correlation of pairwise population distances between the host and parasite (**a**) Average number of differences (Pi), adjusted R^2^ = 0.7127), (**b**) Nei’s Da distance, adjusted R^2^ = 0.6311). Both regressions were significant at P < 0.005. Grey dashed line represents 95% regression confidence interval.

**Figure 6:**
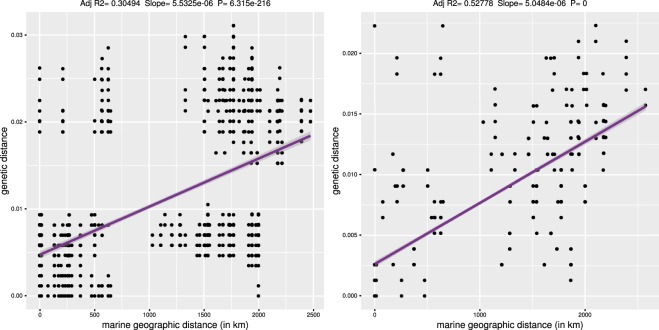
Correlation of pairwise individual genetic distances (computed with Tamura Nei 83 model) and geographic distances in host and parasite. (**a**) Host (Mantel test, observed r = 0.5, P = 0.001), (**b**) Parasite (Mantel test, observed r = 0.7, P = 0.001).

Regression statistics of pairwise population distances of the host and parasite showed very high level of co-diversification (Fig. 5). The slope of regression also suggests that the accumulation of genetic diversity with distance is higher in the host than in the parasite. Mantel test comparisons of individual genetic and geographic distances produced significant values in both the host and the parasite, which is pointing to IBD affecting their genetic structure (Fig. 6). There were a few population samples deviating from this pattern in both the host and parasite, showing high genetic differentiation at a small geographical scale (upper left quadrant in the plots, Fig. 6). These data points represent Sardinian population, which was genetically diverse also in other analyses (Fig. 2, Table 1). Mantel test results calculated without the distant Greek and Cyprus populations produced significant values for both host and parasite datasets (Supplementary Table S5). Despite the trend for co-diversification of the host and parasite across the Mediterranean identified in correlation analyses and in summary statistics, no pattern of congruency between individual haplotypes, or haplotype clusters, was found by mapping host genetic clusters to the parasite network in Fig. 2. Dicyemid samples bearing the same haplotypes can be associated with multiple host genetic clusters.

In the case of parasites, we observed higher molecular variance between populations than within populations in AMOVA (AMOVA: 76% variance between populations compared to 24% within populations, p = 0.001, Supplementary Table S6). Contrary to parasites, hosts showed similar levels of variance between and within populations (AMOVA: 49% variance between populations and 51% within populations, p = 0.001). This result is pointing to a higher degree of population fragmentation in the parasite in the Mediterranean area.

## Discussion

The gross picture of the genetic diversity of *S*. *officinalis* across the Mediterranean Sea supports the view, already indicated by the previous studies^20–22^, that, probably due to a low dispersion capability, this organism displays a high degree of genetic diversification among the geographically distant populations. A new strong evidence for this view was obtained by including the samples from Sardinian coast, which turned to be an area with the highest degree of diversity, possibly representing a contact zone between the East and West populations (Figs 2 and 3). These findings demonstrate that rather than a continuous population across the Mediterranean Sea, dominantly shaped by IBD, *S*. *officinalis* forms a complex structure with different degree of genetic exchange among the subpopulations. In addition, we were able to demonstrate the assumed incongruencies between the host and parasite genetic structures within local populations.

While the majority of samples were collected from the central area of the Mediterranean Sea (i.e. Adriatic, Ligurian and Tyrrhenian Seas), the inclusion of several distant localities (Atlantic coast of Portugal, Greek coast and Cyprus) allowed for assessing the diversity along the whole West-East axis of the Mediterranean Sea. At this large perspective, the distant localities are genetically completely disconnected and do not share common haplotypes (e.g. Portugal vs. Adriatic vs. Cyprus). Although the number of samples is low for the east localities (Greece + Cyprus), this picture is consistent with the biological predispositions of *S. officinali*s, which is incapable of long distance dispersal, and with the previous genetic studies^20–22^.

An interesting picture was obtained when we compared genetic composition of the Mediterranean Sea area and the Atlantic coast of Portugal. Based on the previous studies we should expect one of the following patterns. First, the AO front serves as a major barrier, causing disconnection between the two adjacent areas. Such picture was retrieved for many organisms (for review see^54^). Second, the potential of the AO front as major determinant of population structure is suppressed by biological traits of the organisms. In such case we would expect to see either mixed population across the whole area (large dispersal capability) or strong fragmentation which would “hide” the effect of any physical barrier. However, in our data, we did not see any of these two possibilities. Instead, we found two distant genetic lineages covering disjunct geographic areas but overlapping at the Sardinian coast. In fact, the Sardinian samples proved to be genetically the most diverse. As shown in the haplotype network (Fig. 2), Sardinian samples included several haplotypes from the “Atlantic” lineage (cluster 1), several haplotypes shared with the Croatia-Italian samples (cluster 3) and even an isolated, genetically unique sample (cluster 2). In the broader analysis, which included additional *S. officinalis* publicly available sequences, the Sardinian coast seem to be located on a high diversity zone caused by an overlap of several genetic clusters (depicted in Fig. 3 and Supplementary Fig. S3). Similarly to the *Sepia*, we found several unrelated haplotypes also in the Sardinian dicyemid population (nodes in dark blue, Fig. 2). Furthermore, the positively significant results of Tajima’s D statistics found for this dicyemid population (Table 1) can be interpreted as a sign of past admixture between several lineages (or a bottleneck, which, however seems less probable given the distribution of Sardinian haplotypes in the network).

This scenario suggests a prominent position of the Sardinian coast as a determinant of genetic diversity, independent on the known oceanographic factors (i.e. the currents and fronts). While the current data do not allow for identification of this spot as a contact zone or source of the genetic diversity, it is interesting to note that similar picture of the diversity at Sardinian coast was obtained for the octopus^55^. Although this octopus study was limited to Sardinia, and the diversity could not therefore be compared to other localities, the obtained pattern strongly suggests presence of two distant genetic lineages (Fig. 1 in^55^). It might also be relevant to mention that in their analysis on connectivity among various Mediterranean areas, Andrello *et al*.^56^, although working with biologically very different model, detected Sardinian coasts as the localities with the highest “betweenness centrality”.

The comparison between the host and the parasite population structures reveals two conspicuous patterns. First, there is no straightforward correspondence between the genetic origin of the host and the parasite, and second, the overall degree of genetic diversification is lower in the parasite than in the host (e.g. correlations in Fig. 4). Usage of Mantel tests on mtDNA data was shown to have limitations, in particular IBD is inferred erroneously when distinct regional populations are pooled^57^. However, we believe this was not the case in our dataset. Based on the lack of diversity in 18S dicyemid data and the congruent pattern of increased local diversity in Sardinia for both the host and its parasite we believe that results of the correlations were not affected by artificial pooling of spatially subdivided populations. To increase the power of IBD tests it is recommended to complement the analyses with multilocus nuclear datasets to increase power of the test^57^. Addition of such markers, despite difficult for the small bodied parasites nested in host tissue, would be highly beneficial for future studies.

In parallel to the processes shaping the global diversity and distribution of the haplotypes (influx of mutations, mixing by migration), the local diversities might be affected by demographic processes. Particularly, parasites may regularly undergo bottlenecks removing considerable portion of the diversity. In our data, a convincing example is provided by the best sampled population from the Adriatic Sea between the Croatian and Italian coasts. The host sample from the Croatian coast is genetically diversified, encompassing 11 different haplotypes, which are evenly distributed (1–4 individuals per haplotype) and most of them are shared with the host samples from the Italian coast (Fig. 2). In contrast, most of their parasites share a unique centrally located haplotype and the rest of the population forms a typical star-like pattern. None of these haplotypes is shared with the parasites from other localities, although a related haplotype was sampled from the Italian coast in Liguria (depicted in light blue in Fig. 2). Ligurian and Adriatic coast of Italy are in this area only three hours by car drive away making it not entirely impossible to sell freshly caught cephalopods on the other coast. Although this practice seems not to be wide spread, we paid particular attention to verification of the geographic origins of the purchased samples with the retailers to rule out such possible mixing. Moreover, in respect to the results of the presented analyses, it is important that any possible misidentification of the geographic origin in these close localities would not affect the revealed patterns.

The observed haplotype pattern, typical for the expansion after bottleneck, is also accompanied by the lowest (most negative) values of the neutrality tests statistics (Table 1). Although the values were statistically significant only for one of the tests (Fu’s Fs) and could be alternatively interpreted as a sign of purifying selection^44^, the whole picture (i.e. the haplotype arrangement and the negative values of the statistics) strongly suggest a bottleneck followed by an expansion. This view is also well compatible with the narrower geographic distribution of the parasite’s eastern Adriatic cluster (exclusively Croatian Coast, with the exception of the single Ligurian haplotype) in comparison with the host’s genetic cluster 3, which encompasses the Croatian as well as the Italian and Sardinian samples, often with shared haplotypes. The resulting scenario thus includes a diversified host population, genetically interconnected between the Croatian and Italian coasts, and a recent recolonisation of the local Croatian population with a single genetic lineage of the parasite (i.e. bottleneck with early stage of the following diversification). This replacement/recolonisation scenario is also well compatible with the high degree of the incongruence between the geography-genetic patterns in the host and the parasites (denoted by stars in the Fig. 2).

Considering possible bias of the population statistics due to the small sample sizes for some localities and a potentially low power of Mantel tests and mtDNA to uncover IBD patterns^57^, we use these statistical parameters rather as an accessory evidence while the main population patterns are derived from the haplotype networks. When summarized, these patterns show that at the big scale, both the hosts and the parasites are strongly diversified and their populations are genetically rather fragmented. This is documented not only by the lack of shared haplotypes among the distant localities, but also by considerable genetic distances among the haplotypes (and many missing haplotypes). In this sense, the character of the parasite’s genetic structure reflects the basic fragmentation and diversity of the host’s populations. At the finer (i.e. local) scale, the parasite’s genetics/geography pattern only partially reflects the distribution of the host. This shows that in local populations the extinctions and replacements take place regularly, leading to the genealogical incongruencies between the hosts and the parasites, and to the decrease of genetic diversity in the parasite. This scenario of reduced genetic diversity is in line with assumptions made by Maze-Guilmo *et al*.^19^ for hermaphroditic parasites with an asexual stage, which seems to be a common mode of reproduction in all dicyemids^23^.

## Supplementary information

Supplementary Information
