# Supplementary material for: Population co-divergence in common cuttlefish (Sepia officinalis) and its dicyemid parasite in the Mediterranean Sea
Source: Sci Rep. 2019 Oct 4;9:14300. doi: 10.1038/s41598-019-50555-9 (PMC6778094; doi:10.1038/s41598-019-50555-9)
Supplement: Supplementary file 1 — Supplementary Information [file 41598_2019_50555_MOESM1_ESM.docx]

Population co-divergence in common cuttlefish (*Sepia officinalis*) and its dicyemid parasite in the Mediterranean Sea

Drábková Marie, Nikola Jachníková, Tomáš Tyml, Hana Sehadová, Oleg Ditrich, Eva Myšková, Václav Hypša, Jan Štefka

**Dicyemid COI primer design**

DNA was extracted with QIAamp DNA Micro Kit (QIAGEN) from a small piece of cuttlefish renal tissue sample stored in ethanol . DNA was sent to GENECORE (Heidelberg) sequencing center for library preparation and sequencing. Sample was sequenced on one lane of Illumina MiSeq in PE mode. Reads were assembled into contigs with Trinity (Grabherr et al., 2011). Publicly available COI sequences of dicyemids were used as a query in local BLAST search (Altschul et al., 1990) of a local database created of contigs assembled in draft transcriptome. Sequence with the best hit was used for primer design in Geneious software. Multiple sets of designed primers were custom made in Sigma-Aldrich and their functionality was tested by PCR amplification on selected samples. PCR conditions were further optimized by sets of gradient PCRs.

References:

Grabherr MG, Haas BJ, Yassour M, et al. Full-length transcriptome assembly from RNA-Seq data without a reference genome. *Nat Biotechnol*. 2011;29(7):644-52. Published 2011 May 15. doi:10.1038/nbt.1883

Altschul, S.F., Gish, W., Miller, W., Myers, E.W. & Lipman, D.J. (1990) "Basic local alignment search tool." J. Mol. Biol. 215:403-410. [PubMed](https://www.ncbi.nlm.nih.gov/pubmed/2231712?dopt=Citation)

**Table S1: Sampling locations**

| Supplementary table S1: Sampling locations | | | | | | |  |
| --- | --- | --- | --- | --- | --- | --- | --- |
| sample | name in dataset | locality | sea | code | sequenced specimens | | |
|  |  |  |  |  | host COI | diciemid COI | dicyemid 18S |
| SOGT1H | SOGT1H | Greece | Aegean | GA |  | 1 | 1 |
| SOGT2H | SOGT2H | Greece | Aegean | GA | 1 | 1 | 1 |
| SOIN1H | SOIN1H | Italy, Napoli | Tyrrhenian | IN |  | 1 | 1 |
| SOIN2H | SOIN2H | Italy, Napoli | Tyrrhenian | IN | 1 | 1 |  |
| SOIT1H | SOIT1H | Italy, Sardinia, Oristano | Tyrrhenian | IS | 1 | 1 |  |
| SOIT2H | SOIT2H | Italy, Sardinia, Oristano | Tyrrhenian | IS | 1 | 1 |  |
| SOIT3H | SOIT3H | Italy, Sardinia, Oristano | Tyrrhenian | IS | 1 | 1 | 1 |
| SOIT4H | SOIT4H | Italy, Sardinia, Oristano | Tyrrhenian | IS | 1 | 1 |  |
| SOIT5H | SOIT5H | Italy, Sardinia, Oristano | Tyrrhenian | IS | 1 | 1 |  |
| SOIC1H | SOIC1H | Italy, Sardinia, Cagliari | Tyrrhenian | IS | 1 | 1 |  |
| SOIC2H | SOIC2H | Italy, Sardinia, Cagliari | Tyrrhenian | IS | 1 | 1 |  |
| SOIC3H | SOIC3H | Italy, Sardinia, Cagliari | Tyrrhenian | IS | 1 | 1 |  |
| SOIC4H | SOIC4H | Italy, Sardinia, Cagliari | Tyrrhenian | IS |  |  |  |
| SOIC5H | SOIC5H | Italy, Sardinia, Cagliari | Tyrrhenian | IS | 1 |  |  |
| SOPP1H | SOPP1H | Portugal, Porto | Atlantic | PT | 1 |  |  |
| SOPP2H | SOPP2H | Portugal, Porto | Atlantic | PT | 1 |  |  |
| SOPM3H | SOPM3H | Portugal, Porto | Atlantic | PT | 1 |  |  |
| SOPM4H | SOPM4H | Portugal, Porto | Atlantic | PT | 1 |  |  |
| SOPM5H | SOPM5H | Portugal, Porto | Atlantic | PT | 1 |  |  |
| SOPM6H | SOPM6H | Portugal, Porto | Atlantic | PT | 1 |  |  |
| SOPM7H | SOPM7H | Portugal, Porto | Atlantic | PT | 1 |  |  |
| SOPV8H | SOPV8H | Spain, Vigo | Atlantic | ST | 1 |  |  |
| SOIS3H | SOIS3H | Italy, La Spezia | Ligurian | IL | 1 |  |  |
| SOIS4H | SOIS4H | Italy, La Spezia | Ligurian | IL | 1 | 1 |  |
| SOIG5H | SOIG5H | Italy, Gaeta | Tyrrhenian | IT | 1 |  |  |
| SOIG6H | SOIG6H | Italy, Gaeta | Tyrrhenian | IT | 1 |  |  |
| SOIG7H | SOIG7H | Italy, Gaeta | Tyrrhenian | IT | 1 | 1 |  |
| SOIG8H | SOIG8H | Italy, Gaeta | Tyrrhenian | IT | 1 | 1 |  |
| SOIV9H | SOIV9H | Italy, Vieste | Adriatic | IA | 1 |  |  |
| SOIV10H | SOIV10H | Italy, Vieste | Adriatic | IA | 1 | 1 | 1 |
| SOIP11H | SOIP11H | Italy, Pescara | Adriatic | IA | 1 | 1 |  |
| SOIP12H | SOIP12H | Italy, Pescara | Adriatic | IA | 1 | 1 |  |
| SOIP13H | SOIP13H | Italy, Pescara | Adriatic | IA | 1 | 1 |  |
| SOIR11H | SOIR11H | Italy, Rimini | Adriatic | IA | 1 |  | 1 |
| SOIR12H | SOIR12H | Italy, Rimini | Adriatic | IA | 1 |  |  |
| SOIR13H | SOIR13H | Italy, Rimini | Adriatic | IA | 1 |  |  |
| SOV1H | SOCV1H | Croatia, Veruda-Pula | Adriatic | CA |  | 1 |  |
| SOV2H | SOCV2H | Croatia, Veruda-Pula | Adriatic | CA | 1 | 1 |  |
| SOV3H | SOCV3H | Croatia, Veruda-Pula | Adriatic | CA | 1 | 1 |  |
| SOV4H | SOCV4H | Croatia, Veruda-Pula | Adriatic | CA | 1 | 1 |  |
| SOV5H | SOCV5H | Croatia, Veruda-Pula | Adriatic | CA | 1 | 1 | 1 |
| SOV6H | SOCV6H | Croatia, Veruda-Pula | Adriatic | CA | 1 | 1 | 1 |
| SOV7H | SOCV7H | Croatia, Veruda-Pula | Adriatic | CA | 1 | 1 |  |
| SOV8H | SOCV8H | Croatia, Veruda-Pula | Adriatic | CA |  | 1 | 1 |
| SOV9H | SOCV9H | Croatia, Veruda-Pula | Adriatic | CA |  | 1 | 1 |
| SOV10H | SOCV10H | Croatia, Veruda-Pula | Adriatic | CA |  | 1 | 1 |
| SOV11H | SOCV11H | Croatia, Veruda-Pula | Adriatic | CA |  | 1 | 1 |
| SOV13H | SOCV13H | Croatia, Veruda-Pula | Adriatic | CA | 1 | 1 | 1 |
| SOV14H | SOCV14H | Croatia, Veruda-Pula | Adriatic | CA | 1 | 1 |  |
| SOT1H | SOCT1H | Croatia, Trogir | Adriatic | CA | 1 | 1 | 1 |
| SOT2H | SOCT2H | Croatia, Trogir | Adriatic | CA | 1 | 1 |  |
| SOT3H | SOCT3H | Croatia, Trogir | Adriatic | CA |  | 1 |  |
| SOP1H/SEP1H | SOCA1H | Croatia, Pula | Adriatic | CA | 1 | 1 |  |
| SOP2H | SOCA2H | Croatia, Pula | Adriatic | CA | 1 |  |  |
| SOP3H | SOCA3H | Croatia, Pula | Adriatic | CA | 1 | 1 | 1 |
| SOP4H | SOCA4H | Croatia, Pula | Adriatic | CA | 1 | 1 | 1 |
| SOP5H | SOCA5H | Croatia, Pula | Adriatic | CA | 1 | 1 |  |
| SOP6H | SOCA6H | Croatia, Pula | Adriatic | CA | 1 | 1 |  |
| SOP7H | SOCA7H | Croatia, Pula | Adriatic | CA | 1 | 1 | 1 |
| SOP8H | SOCA8H | Croatia, Pula | Adriatic | CA | 1 | 1 |  |
| SOP9H | SOCA9H | Croatia, Pula | Adriatic | CA | 1 | 1 | 1 |
| SOP10H | SOCA10H | Croatia, Pula | Adriatic | CA | 1 | 1 |  |
| SOP11H | SOCA11H | Croatia, Pula | Adriatic | CA | 1 | 1 |  |
| SOP12H | SOCA12H | Croatia, Pula | Adriatic | CA | 1 | 1 |  |
| SOP13H | SOCA13H | Croatia, Pula | Adriatic | CA |  | 1 | 1 |
| SNC1H | SOKL1H | Cyprus, Limassol | East Mediterranean | EM | 1 | 1 | 1 |
| SNC2H | SOKL2H | Cyprus, Limassol | East Mediterranean | EM | 1 | 1 | 1 |
| SNC3H | SOKL3H | Cyprus, Limassol | East Mediterranean | EM | 1 | 1 | 1 |
| SOI1H | SOIE1H | Italy, La Spezia | Ligurian | IL | 1 |  |  |
| SOI2H | SOIE2H | Italy, La Spezia | Ligurian | IL | 1 |  | 1 |
| reference seq | SOXX1H | AB2401555 |  |  | 1 |  |  |
|  |  |  |  | SUM | 61 | 50 | 23 |

**Table S2: PCR conditions and primer sequences**

| Table S2: PCR conditions and primer sequences | | | | | | | |  |
| --- | --- | --- | --- | --- | --- | --- | --- | --- |
| PCR reaction | | | | | recipe | | conditions |  |
| cephalopod host | F1490 (COI) | GGTCAACAAATCATAAAGATATTGG | Folmer 1994 | 20µl reaction 2 µl PCR blue buffer (Top-Bio) 0.2 µl Taq Polymerase (Top-Bio) 1 µl sample 1 µl 5mM forward primer 1 µl 5mM reverse primer 0.5 µl 10mM each dNTPs 14.3 µl H20 | | The PCR protocol involved an initial denaturation period (94 °C for 5 min), 30 cycles of denaturing (92 °C for 1 min), annealing (52 °C for 1 min), and elongation (72 °C for 1 min), and a final elonga-tion period (72 °C for 5 min) | |  |
|  | H7005 (COI) | CCGGATCCACANCRTARTANGTRTCRTG | Hafner 1994 |  |  |  |  |  |
| dicyemid parasite | dic9Fd (COI) | GCTTGTGCWGGBTGAACTCTATATCCWCC | designed in this study |  |  | The PCR protocol involved an initial denaturation period (95 °C for 5 min), 30 cycles of denaturing (92 °C for 1 min), annealing (53 °C for 1 min), and elongation (72 °C for 1 min), and a final elongation period (72 °C for 5 min) | |  |
|  | dic6Rd (COI) | TGYATAARRTAWCGMCGAGGTATHGCWGM | designed in this study |  |  |  |  |  |
|  | F3 (18S) | CGGCTCATTAAATCGGACATAC | Eshragh and Leander 2014 |  |  | The PCR protocol involved an initial denaturation period (94°C for 2 min), 40 cycles of denaturing (94°C for 45 s), annealing (50°C for 45 s), and elongation (72°C for 2 min), and a final elonga-tion period (72°C for 5 min) | |  |
|  | R2 (18S) | CCAACAACCTCACCAAATCATTC | Eshragh and Leander 2014 |  |  |  |  |  |
| sequencing | | | | | sequencing primers | | |  |
| cephalopod host | H7005 | CCGGATCCACANCRTARTANGTRTCRTG | Hafner 1994 | cephCOIin14R | | CCKGTGGGRATKGCAATAATTAT | | designed in this study |
|  |  |  |  |  |  |  |  |  |
|  | F1490 | GGTCAACAAATCATAAAGATATTGG | Folmer 1994 | cephCOIin11F | | CGATGAGAAGGTWTAYWAATAGAACG | | designed in this study |
|  |  |  |  |  |  |  |  |  |
| dicyemid parasite | dic9Fd | GCTTGTGCWGGBTGAACTCTATATCCWCC | designed in this study | - | | - | | - |
|  | dic6Rd | TGYATAARRTAWCGMCGAGGTATHGCWGM | designed in this study | - | | - | | - |

**Table S3: Accesion numbers of sequences used in phylogenetic tree and population network**

| phylogenetic tree |  |  | *Sepia officinalis* population network |  |  |  |
| --- | --- | --- | --- | --- | --- | --- |
| Nautilus | AY557514.1 |  | KF369184.1 | EF416562.1 | EF416326.1 | EF416308.1 |
| Sepia esculenta | NC009690 |  | EF416483.1 | EF416559.1 | EF416322.1 | EF416459.1 |
| Sepia pharaonis | NC021146 |  | EF416482.1 | EF416484.1 | EF416321.1 | EF416348.1 |
| Sepia elegans | AB430404.1 |  | EF416480.1 | EF416476.1 | EF416318.1 | EF416347.1 |
| Sepia lycidas | NC022468 |  | EF416479.1 | EF416415.1 | EF416317.1 | EF416342.1 |
| Sepietta oweniana | EU203144 |  | EF416475.1 | EF416558.1 | EF416314.1 | EF416320.1 |
| Sepiett neglecta | KM517940 |  | EF416478.1 | EF416556.1 | EF416313.1 | EF416307.1 |
| Sepietta obscura | AY293720 |  | EF416553.1 | EF416468.1 | EF416310.1 | EF416311.1 |
| Sepiola rondeleti | AY293720 |  | EF416551.1 | EF416467.1 | EF416309.1 | EF416402.1 |
| Semirossia | NC015425 |  | EF416549.1 | EF416453.1 | EF416306.1 | EF416525.1 |
| Architeuthis | EU735375.1 |  | EF416548.1 | EF416452.1 | EF416546.1 | EF416462.1 |
| Bathyteuthis | NC016423 |  | EF416547.1 | EF416450.1 | EF416534.1 | EF416316.1 |
| Sepia apama | AP013073 |  | EF416544.1 | EF416449.1 | EF416529.1 | EF416312.1 |
| Sepia latimanus | NC022467 |  | EF416542.1 | EF416448.1 | EF416526.1 | EF416335.1 |
| Sepia elegans | KM517937.1 |  | EF416537.1 | EF416445.1 | EF416517.1 | KF369183.1 |
| Sepia elegans | AM293707.1 |  | EF416527.1 | EF416405.1 | EF416434.1 | EF416552.1 |
| Sepia officinalis | AB2401555 |  | EF416463.1 | EF416408.1 | EF416423.1 | EF416533.1 |
|  |  |  | EF416460.1 | EF416424.1 | EF416374.1 | EF416531.1 |
|  |  |  | EF416458.1 | EF416454.1 | EF416315.1 | EF416522.1 |
|  |  |  | EF416444.1 | EF416451.1 | EF416358.1 | EF416521.1 |
|  |  |  | EF416443.1 | EF416447.1 | EF416554.1 | EF416519.1 |
|  |  |  | EF416440.1 | EF416433.1 | EF416550.1 | EF416400.1 |
|  |  |  | EF416438.1 | EF416432.1 | EF416455.1 | EF416543.1 |
|  |  |  | EF416414.1 | EF416431.1 | EF416442.1 | EF416532.1 |
|  |  |  | EF416398.1 | EF416430.1 | EF416441.1 | EF416530.1 |
|  |  |  | EF416396.1 | EF416428.1 | EF416437.1 | EF416524.1 |
|  |  |  | EF416395.1 | EF416427.1 | EF416435.1 | EF416523.1 |
|  |  |  | EF416394.1 | EF416425.1 | EF416390.1 | EF416520.1 |
|  |  |  | EF416393.1 | EF416422.1 | EF416389.1 | EF416515.1 |
|  |  |  | EF416391.1 | EF416421.1 | EF416388.1 | AB193812.1 |
|  |  |  | EF416386.1 | EF416420.1 | EF416382.1 | EF416403.1 |
|  |  |  | EF416385.1 | EF416419.1 | EF416381.1 | EF416401.1 |
|  |  |  | EF416384.1 | EF416418.1 | EF416378.1 | EF416399.1 |
|  |  |  | EF416383.1 | EF416417.1 | EF416369.1 | EF416397.1 |
|  |  |  | EF416380.1 | EF416416.1 | EF416364.1 | EF416387.1 |
|  |  |  | EF416379.1 | EF416413.1 | EF416343.1 | EF416392.1 |
|  |  |  | EF416377.1 | EF416412.1 | EF416340.1 | EF416539.1 |
|  |  |  | EF416376.1 | EF416411.1 | EF416324.1 | EF416528.1 |
|  |  |  | EF416375.1 | EF416409.1 | EF416372.1 | EF416518.1 |
|  |  |  | EF416373.1 | EF416407.1 | EF416370.1 | EF416516.1 |
|  |  |  | EF416371.1 | EF416446.1 | EF416366.1 | EF416514.1 |
|  |  |  | EF416367.1 | EF416429.1 | EF416365.1 | EF416511.1 |
|  |  |  | EF416361.1 | EF416426.1 | EF416363.1 | EF416510.1 |
|  |  |  | EF416360.1 | EF416410.1 | EF416362.1 | EF416508.1 |
|  |  |  | EF416359.1 | EF416406.1 | EF416353.1 | EF416505.1 |
|  |  |  | EF416357.1 | EF416471.1 | EF416352.1 | EF416504.1 |
|  |  |  | EF416356.1 | EF416545.1 | EF416338.1 | EF416501.1 |
|  |  |  | EF416355.1 | EF416540.1 | EF416368.1 | EF416500.1 |
|  |  |  | EF416354.1 | EF416541.1 | EF416461.1 | EF416499.1 |
|  |  |  | EF416351.1 | EF416536.1 | EF416332.1 | EF416497.1 |
|  |  |  | EF416350.1 | EF416538.1 | EF416330.1 | EF416496.1 |
|  |  |  | EF416349.1 | EF416472.1 | EF416325.1 | EF416495.1 |
|  |  |  | EF416346.1 | EF416535.1 | EF416323.1 | EF416494.1 |
|  |  |  | EF416345.1 | EF416474.1 | EF416439.1 | EF416493.1 |
|  |  |  | EF416344.1 | EF416481.1 | EF416436.1 | EF416491.1 |
|  |  |  | EF416341.1 | EF416404.1 | EF416333.1 | EF416490.1 |
|  |  |  | EF416339.1 | EF416561.1 | EF416319.1 | EF416489.1 |
|  |  |  | EF416337.1 | EF416557.1 | EF416477.1 | EF416513.1 |
|  |  |  | EF416336.1 | EF416473.1 | EF416464.1 | EF416512.1 |
|  |  |  | EF416334.1 | EF416470.1 | EF416457.1 | EF416487.1 |
|  |  |  | EF416331.1 | EF416469.1 | EF416456.1 | EF416486.1 |
|  |  |  | EF416329.1 | EF416466.1 | EF416327.1 | EF416509.1 |
|  |  |  | EF416328.1 | EF416465.1 | EF416564.1 | EF416507.1 |
|  |  |  | EF416498.1 | EF416492.1 | EF416563.1 | EF416506.1 |
|  |  |  | EF416503.1 | EF416485.1 | EF416560.1 |  |
|  |  |  | EF416502.1 | EF416488.1 | EF416555.1 |  |
| sequences produced in this study | | | | | | |
|  |  | host COI | dicyemid COI | dicyemid 18S | locality | sea |
|  | SOGT1H |  | MN069283 | MN066350 | Greece | Aegean |
|  | SOGT2H | MN069209 | MN069298 | MN066359 | Greece | Aegean |
|  | SOIN1H |  | MN069297 | MN066360 | Italy, Napoli | Tyrrhenian |
|  | SOIN2H | MN069219 | MN069296 |  | Italy, Napoli | Tyrrhenian |
|  | SOIT1H | MN069208 | MN069286 |  | Italy, Sardinia, Oristano | Tyrrhenian |
|  | SOIT2H | MN069245 | MN069301 |  | Italy, Sardinia, Oristano | Tyrrhenian |
|  | SOIT3H | MN069193 | MN069300 | MN066356 | Italy, Sardinia, Oristano | Tyrrhenian |
|  | SOIT4H | MN069204 | MN069299 |  | Italy, Sardinia, Oristano | Tyrrhenian |
|  | SOIT5H | MN069221 | MN069285 |  | Italy, Sardinia, Oristano | Tyrrhenian |
|  | SOIC1H | MN069216 | MN069295 |  | Italy, Sardinia, Cagliari | Tyrrhenian |
|  | SOIC2H | MN069217 | MN069294 |  | Italy, Sardinia, Cagliari | Tyrrhenian |
|  | SOIC3H | MN069244 | MN069293 |  | Italy, Sardinia, Cagliari | Tyrrhenian |
|  | SOIC4H |  | MN310702 |  | Italy, Sardinia, Cagliari | Tyrrhenian |
|  | SOIC5H | MN069218 |  |  | Italy, Sardinia, Cagliari | Tyrrhenian |
|  | SOPP1H | MN069249 |  |  | Portugal, Porto | Atlantic |
|  | SOPP2H | MN069248 |  |  | Portugal, Porto | Atlantic |
|  | SOPM3H | MN069223 |  |  | Portugal, Porto | Atlantic |
|  | SOPM4H | MN069194 |  |  | Portugal, Porto | Atlantic |
|  | SOPM5H | MN069220 |  |  | Portugal, Porto | Atlantic |
|  | SOPM6H | MN069196 |  |  | Portugal, Porto | Atlantic |
|  | SOPM7H | MN069246 |  |  | Portugal, Porto | Atlantic |
|  | SOPV8H | MN069206 |  |  | Spain, Vigo | Atlantic |
|  | SOIS3H | MN069228 |  |  | Italy, La Spezia | Ligurian |
|  | SOIS4H | MN069230 | MN069287 |  | Italy, La Spezia | Ligurian |
|  | SOIG5H | MN069234 |  |  | Italy, Gaeta | Tyrrhenian |
|  | SOIG6H | MN069227 |  |  | Italy, Gaeta | Tyrrhenian |
|  | SOIG7H | MN069241 | MN069288 |  | Italy, Gaeta | Tyrrhenian |
|  | SOIG8H | MN069242 | MN069289 |  | Italy, Gaeta | Tyrrhenian |
|  | SOIV9H | MN069199 |  |  | Italy, Vieste | Adriatic |
|  | SOIV10H | MN069205 | MN069255 | MN066357 | Italy, Vieste | Adriatic |
|  | SOIP11H | MN069235 | MN069292 |  | Italy, Pescara | Adriatic |
|  | SOIP12H | MN069237 | MN069291 |  | Italy, Pescara | Adriatic |
|  | SOIP13H | MN069229 | MN069290 |  | Italy, Pescara | Adriatic |
|  | SOIR11H | MN069240 | MN310703 | MN066367 | Italy, Rimini | Adriatic |
|  | SOIR12H | MN069238 |  |  | Italy, Rimini | Adriatic |
|  | SOIR13H | MN069231 | MN310704 |  | Italy, Rimini | Adriatic |
|  | SOCV1H |  | MN069275 |  | Croatia, Veruda-Pula | Adriatic |
|  | SOCV2H | MN069225 | MN069276 |  | Croatia, Veruda-Pula | Adriatic |
|  | SOCV3H | MN069226 | MN069282 |  | Croatia, Veruda-Pula | Adriatic |
|  | SOCV4H | MN069195 | MN069261 |  | Croatia, Veruda-Pula | Adriatic |
|  | SOCV5H | MN069239 | MN069260 | MN066358 | Croatia, Veruda-Pula | Adriatic |
|  | SOCV6H | MN069243 | MN069259 | MN066366 | Croatia, Veruda-Pula | Adriatic |
|  | SOCV7H | MN069197 | MN069267 |  | Croatia, Veruda-Pula | Adriatic |
|  | SOCV8H |  | MN069258 | MN066355 | Croatia, Veruda-Pula | Adriatic |
|  | SOCV9H |  | MN069257 | MN066346 | Croatia, Veruda-Pula | Adriatic |
|  | SOCV10H |  | MN069256 | MN066364 | Croatia, Veruda-Pula | Adriatic |
|  | SOCV11H |  | MN069278 | MN066352 | Croatia, Veruda-Pula | Adriatic |
|  | SOCV13H | MN069202 | MN069284 | MN066362 | Croatia, Veruda-Pula | Adriatic |
|  | SOCV14H | MN069201 | MN069273 |  | Croatia, Veruda-Pula | Adriatic |
|  | SOCT1H | MN069224 | MN069262 | MN066363 | Croatia, Trogir | Adriatic |
|  | SOCT2H | MN069215 | MN069270 |  | Croatia, Trogir | Adriatic |
|  | SOCT3H |  | MN069271 |  | Croatia, Trogir | Adriatic |
|  | SOCA1H | MN069247 | MN069268 |  | Croatia, Pula | Adriatic |
|  | SOCA2H | MN069212 |  |  | Croatia, Pula | Adriatic |
|  | SOCA3H | MN069213 | MN069279 | MN066345 | Croatia, Pula | Adriatic |
|  | SOCA4H | MN069222 | MN069265 | MN066365 | Croatia, Pula | Adriatic |
|  | SOCA5H | MN069192 | MN069274 |  | Croatia, Pula | Adriatic |
|  | SOCA6H | MN069214 | MN069264 |  | Croatia, Pula | Adriatic |
|  | SOCA7H | MN069236 | MN069272 | MN066361 | Croatia, Pula | Adriatic |
|  | SOCA8H | MN069191 | MN069263 |  | Croatia, Pula | Adriatic |
|  | SOCA9H | MN069200 | MN069280 | MN066354 | Croatia, Pula | Adriatic |
|  | SOCA10H | MN069190 | MN069269 |  | Croatia, Pula | Adriatic |
|  | SOCA11H | MN069211 | MN069281 |  | Croatia, Pula | Adriatic |
|  | SOCA12H | MN069203 | MN069277 |  | Croatia, Pula | Adriatic |
|  | SOCA13H |  | MN069266 | MN066353 | Croatia, Pula | Adriatic |
|  | SOKL1H | MN069232 | MN069254 | MN066347 | Cyprus, Limassol | East Mediterranean |
|  | SOKL2H | MN069233 | MN069253 | MN066348 | Cyprus, Limassol | East Mediterranean |
|  | SOKL3H | MN069207 | MN069252 | MN066349 | Cyprus, Limassol | East Mediterranean |
|  | SOIE1H | MN069198 |  |  | Italy, La Spezia | Ligurian |
|  | SOIE2H | MN069210 |  | MN066351 | Italy, La Spezia | Ligurian |
| not in population network | SOIM1H | MN069250 |  |  | Italy, Sicily, Marsala | Tyrrhenian |
| not in population network | SOIM2H | MN069251 |  |  | Italy, Sicily, Marsala | Tyrrhenian |

**Table S4: Fst values computed for downsampled dataset with 5 samples maximum per population. Light grey and top row values for host populations, dark grey and left column values for parasites.**

|  |  |  |  |  |  |  |
| --- | --- | --- | --- | --- | --- | --- |
| **Fst** | Croatia Adriatic (5) | Sardinia (5) | Italy Tyrrhenian (5) | Portugal (5) | Italy Adriatic (5) | Cyprus (3) |
| Croatia Adriatic (5) |  | 0.445 | 0.357 | 0.849 | *-0.034* | 0.826 |
| Sardinia (5) | 0.438 |  | *0.355* | 0.217 | 0.457 | 0.549 |
| Italy Tyrrhenian (5) | 0.725 | 0.474 |  | 0.846 | 0.415 | 0.873 |
| Portugal - | - | - | - |  | 0.857 | 0.894 |
| Italy Adriatic (4) | 0.597 | *0.381* | *0.121* | - |  | 0.852 |
| Cyprus (3) | 0.913 | 0.573 | 0.822 | - | 0.777 |  |
|  |  |  |  |  |  |  |
|  | non significant values in italics (level 0.05) | | | |  |  |

| **general overview** downsampled populations | | Portugal | Sardinia | | Italy Tyrrhenian | Italy Adriatic | Croatia Adriatic | |
| --- | --- | --- | --- | --- | --- | --- | --- | --- |
| number of sequences |  | 5 | 5 | 5 | 5 | 5 | 5 | 5 |
| polymorphic (segregating) sites | S | 6 | 24 | 15 | 8 | 7 | 9 | 2 |
| number of haplotypes | h | 4 | 5 | 3 | 3 | 5 | 4 | 3 |
| haplotype diversity | Hd | 0.161 | 1 | 0.7 | 0.7 | 1 | 0.9 | 0.8 |
| nucleotide diversity | Pi | 0.003 | 0.016 | 0.011 | 0.004 | 0.004 | 0.004 | 0.013 |
| Theta per sequence (from S) | Theta-W | 2.9 | 11.5 | 7.2 | 3.8 | 3.4 | 4.3 | 1 |
| average number of pairwise differences | k | 2.6 | 12.8 | 8.8 | 3.2 | 3.4 | 3.8 | 1 |
| Raggedness | r | 0.09 | 0.1 | 0.23 | 0.23 | 0.08 | 0.15 | 0.36 |
| Ramos-Onsins and Rozas | R2 | 0.217 | 0.214 | 0.281 | 0.341 | 0.167 | 0.184 | 0.25 |
| Fu's | Fs | -0.567 | -0.019 | 3.51 | 1.458 | -2.004 | 0.051 | -0.475 |
| Fu and Li | D* | -0.668 | 0.826 | 1.625 | -1.174 | 0.083 | -0.855 | 0.243 |
| Fu and Li | F* | -0.692 | 0.888 | 1.731 | -1.23 | 0.086 | -0.899 | 0.239 |
| Tajima's D | D | -0.668 | 0.826 | 1.625 | -1.174 | 0.083 | -0.855 | 0.243 |
|  |  |  | legend | | | |  |  |
|  |  |  | host | | parasite | |  |  |
|  |  |  |  |  |  |  |  |  |

| **Table S5: Results of mantel test for testing of relationship between geographical and genetic distance of samples without samples originating from Greece and Cyprus because of low sample size.**   \| host: sepia \|  \|  \| \| --- \| --- \| --- \| \|  \|  \|  \| \| Monte-Carlo test \|  \|  \| \| Call: mantel.randtest(m1 = GEOdistIndividuals3, m2 = AdeGenDist) \| \| \| \| Observation: 0.6351138 \|  \|  \| \| Based on 999 replicates \|  \|  \| \| Simulated p-value: 0.001 \|  \|  \| \| Alternative hypothesis: greater \|  \|  \| \|  \|  \|  \| \| Std.Obs Expectation Variance \|  \|  \| \| 10.542671150 0.002813014 0.003597047 \|  \|  \|   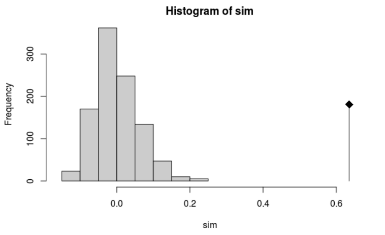 | | | |
| --- | --- | --- | --- | --- | --- | --- | --- | --- | --- | --- | --- | --- | --- | --- | --- | --- | --- | --- | --- | --- | --- | --- | --- | --- | --- | --- | --- | --- | --- | --- | --- | --- | --- | --- | --- | --- |
|  |  |  |  |
|  |  |  |  |
|  |  |  |  |
|  |  |  |  |
|  |  |  |  |
|  |  |  |  |
|  |  |  |  |
|  |  |  |  |
|  |  |  |  |
|  |  |  |  |
|  |  |  |  |
|  |  |  |  |
|  |  |  |  |
|  |  |  |  |
|  |  |  |  |
|  |  |  |  |
| parasite:dicyemids |  |  |  |
|  |  |  |  |
| Monte-Carlo test |  |  |  |
| Call: mantel.randtest(m1 = GEOdistIndividuals3, m2 = AdeGenDist) | | |  |
| Observation: 0.6765353 |  |  |  |
| Based on 999 replicates |  |  |  |
| Simulated p-value: 0.001 |  |  |  |
| Alternative hypothesis: greater |  |  |  |
|  |  |  |  |
| Std.Obs Expectation Variance |  |  |  |
| 8.5430874848 -0.0008411328 0.0062868119 |  |  |  |


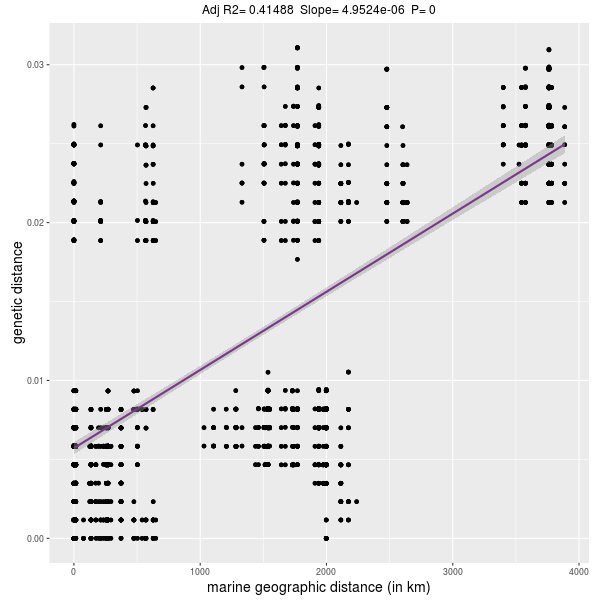

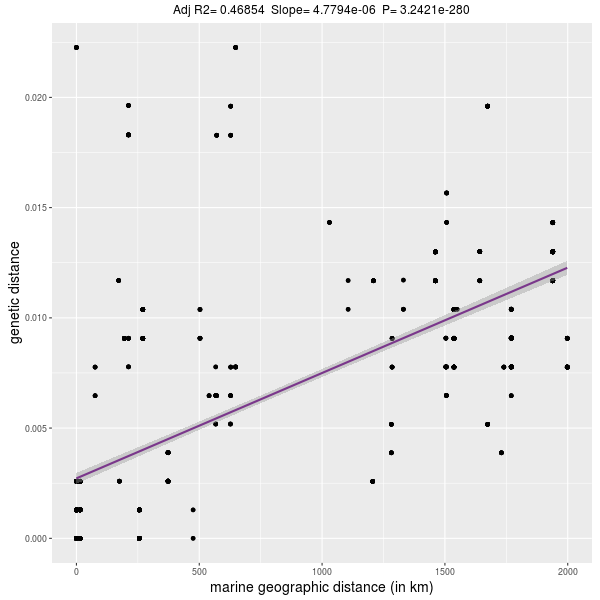


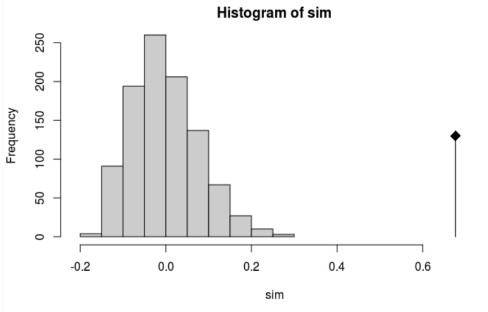


**Table S6: Results of AMOVA analysis.**


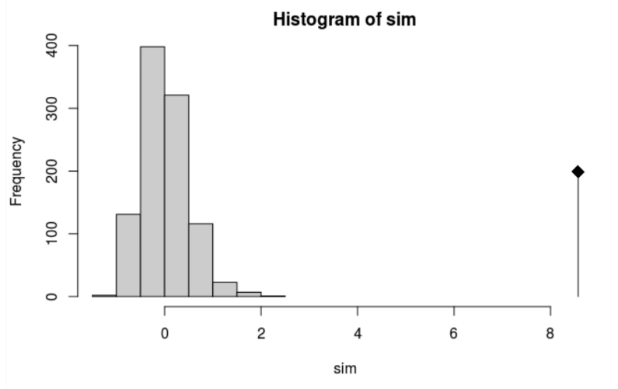


| host: sepia |
| --- |
| $call |
| ade4::amova(samples = xtab, distances = xdist, structures = xstruct) |
| $results |
| Df Sum Sq Mean Sq |
| Between samples 7 446.1323 63.73319 |
| Within samples 53 253.0808 4.77511 |
| Total 60 699.2131 11.65355 |
| $componentsofcovariance |
| Sigma % |
| Variations Between samples 8.574625 **64.23068** |
| Variations Within samples 4.775110 **35.76932** |
| Total variations 13.349735 100.00000 |
| $statphi |
| Phi |
| Phi-samples-total 0.6423068 |
|  |
| Monte-Carlo test |
| Call: as.randtest(sim = res, obs = sigma[1]) |
| Observation: 8.574625 |
| Based on 999 replicates |
| Simulated p-value: 0.001 |
| Alternative hypothesis: greater |
| Std.Obs Expectation Variance |
| 18.03441878 0.00839569 0.22561870 |

| host: sepia without Portugal |
| --- |
| $call |
| ade4::amova(samples = xtab, distances = xdist, structures = xstruct) |
| $results |
| Df Sum Sq Mean Sq |
| Between samples 5 238.6453 47.729060 |
| Within samples 46 231.7778 5.038647 |
| Total 51 470.4231 9.223982 |
| $componentsofcovariance |
| Sigma % |
| Variations Between samples 5.780993 53.43055 |
| Variations Within samples 5.038647 46.56945 |
| Total variations 10.819641 100.00000 |
| $statphi |
| Phi |
| Phi-samples-total 0.5343055 |
|  |
| Monte-Carlo test |
| Call: as.randtest(sim = res, obs = sigma[1]) |
| Observation: 5.780993 |
| Based on 999 replicates |
| Simulated p-value: 0.001 |
| Alternative hypothesis: greater |
| Std.Obs Expectation Variance |
| 14.208774148 0.007951067 0.165080682 |


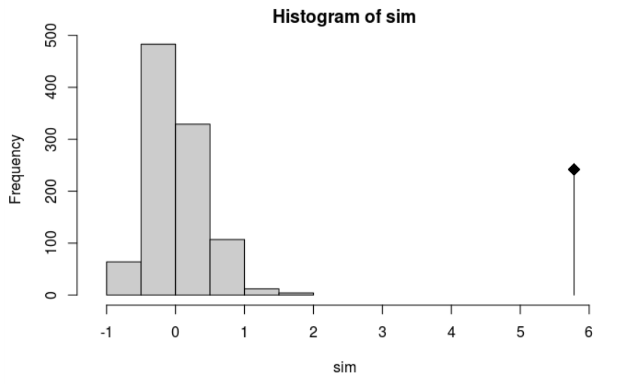


| parasites: dicyemids |
| --- |
| $call |
| ade4::amova(samples = xtab, distances = xdist, structures = xstruct) |
| $results |
| Df Sum Sq Mean Sq |
| Between samples 5 173.231 34.646190 |
| Within samples 44 122.969 2.794751 |
| Total 49 296.200 6.044898 |
| $componentsofcovariance |
| Sigma % |
| Variations Between samples 4.983016 **64.06744** |
| Variations Within samples 2.794751 **35.93256** |
| Total variations 7.777767 100.00000 |
| $statphi |
| Phi |
| Phi-samples-total 0.6406744 |
|  |
| Monte-Carlo test |
| Call: as.randtest(sim = res, obs = sigma[1]) |
| Observation: 4.983016 |
| Based on 999 replicates |
| Simulated p-value: 0.001 |
| Alternative hypothesis: greater |
| Std.Obs Expectation Variance |
| 16.878530710 0.005642233 0.086962349 |


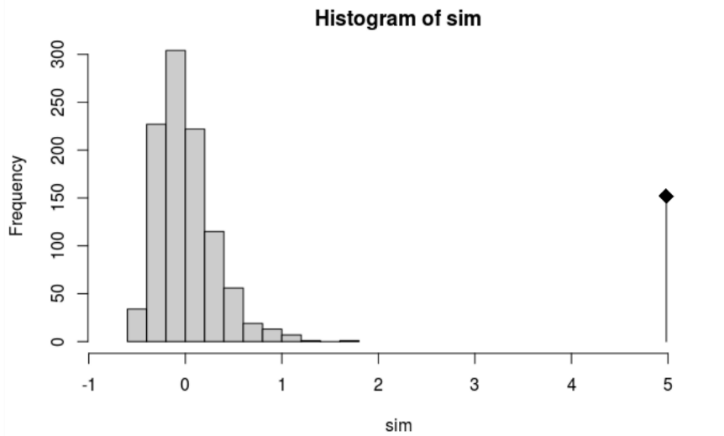


**Supplementary Figure S1: Maximum likelihood tree based on COI gene constructed in IQ-TREE (model auto selected-TIM2+F+I+G4, 1533bp). Pink bubble denotes the position of two sequences with 10% difference, not included in population analyses.**

**Node supports expressed as aLRT/UFB.
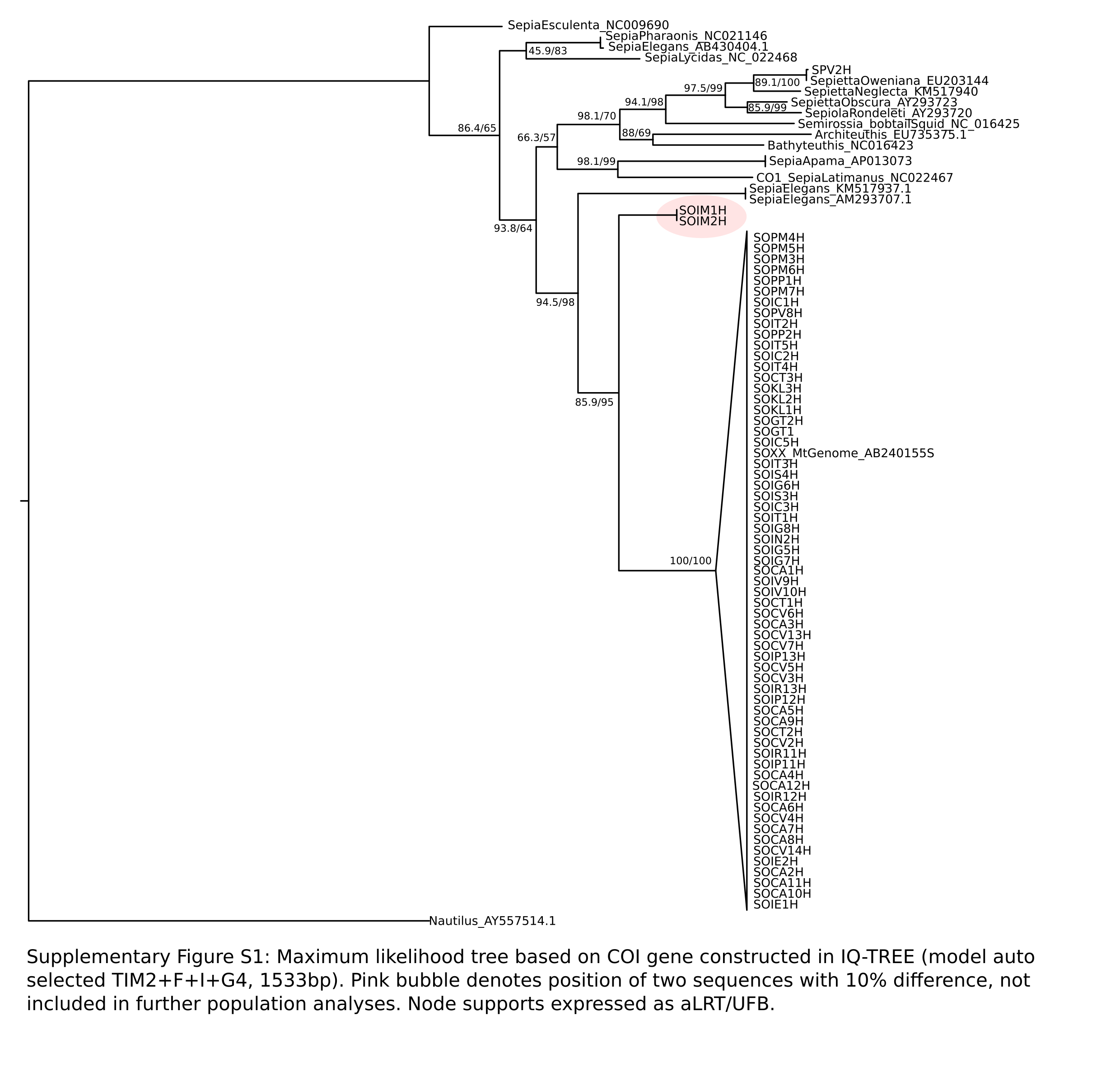
**

**Supplementary Figure S2: Principal component analysis computed in R of both parasite (dicyemid) and its host (Sepia).**


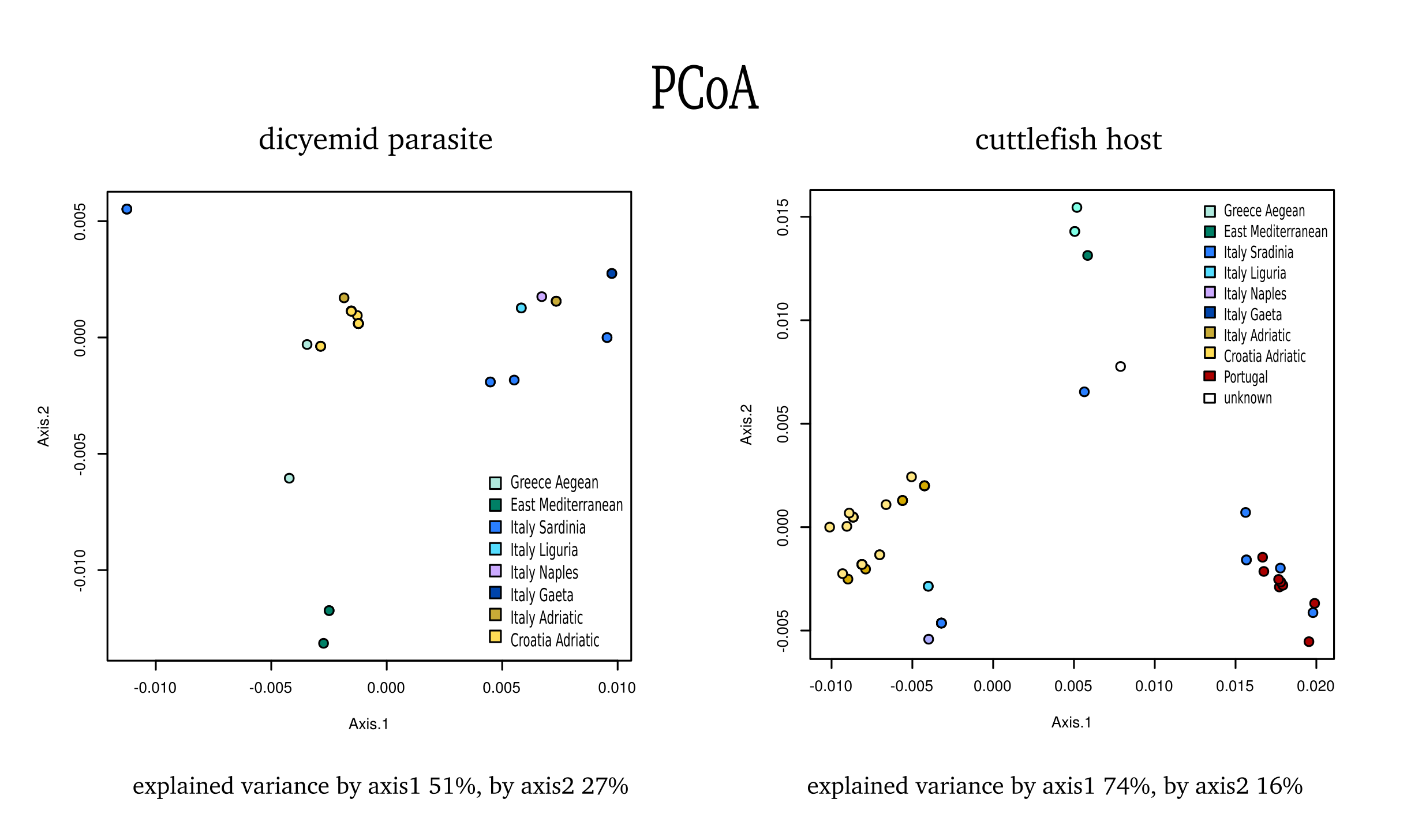


**Supplementary Figure S3: Distribution of *Sepia officinalis* genetic clusters in the Mediterranean Sea focusing on the diversity captured on Sardinia.**

**
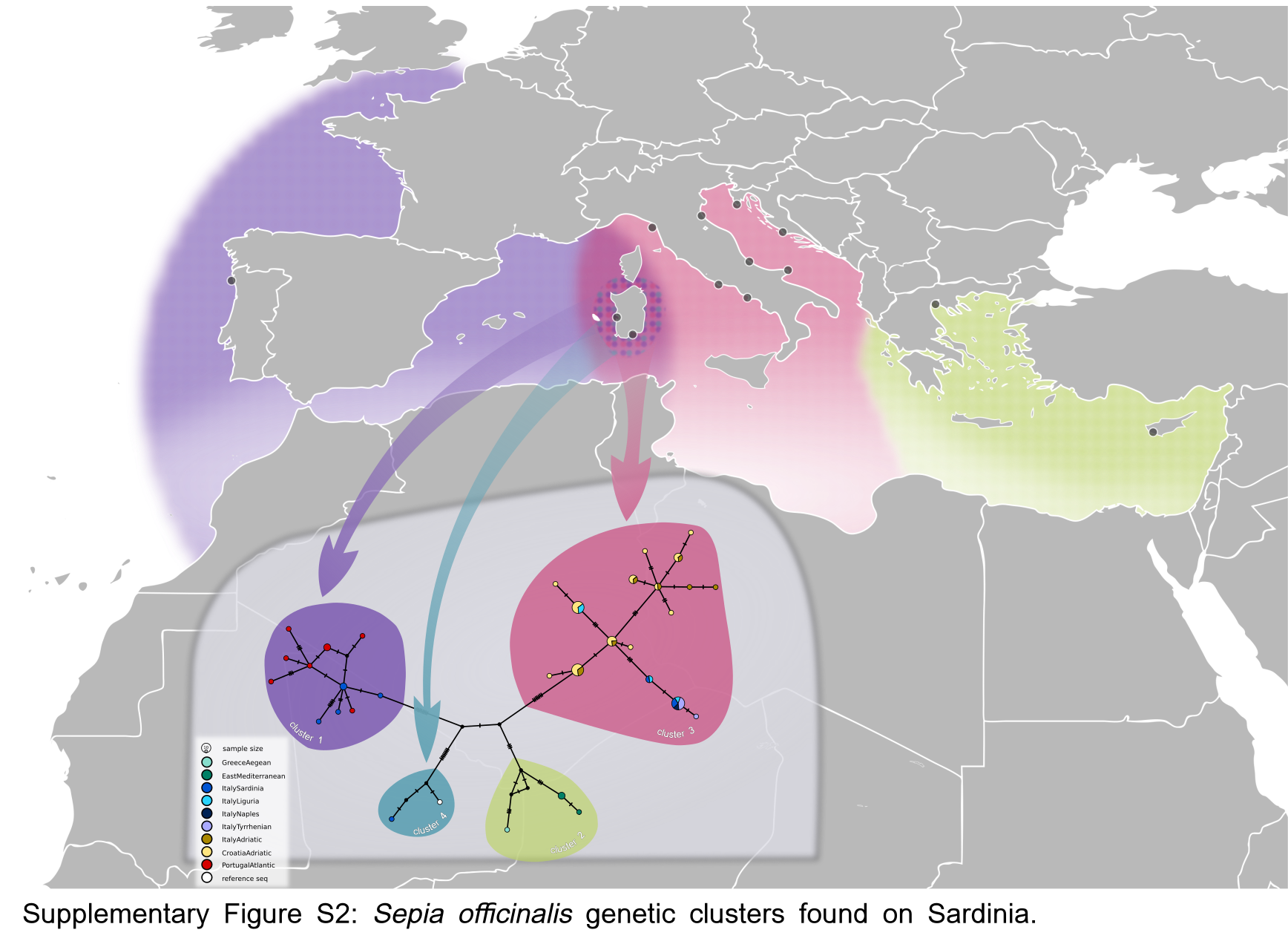
**

**R code used for computation and plotting of the Mantel test and isolation by distance**

#Rmd notebook blocks

```{r}

library("apex")

library("adegenet")

library("pegas")

library("mmod")

library("poppr")

library("seqinr")

library("Matrix")

library("ggplot2")

```

```{r}

setwd("/home/marie/Desktop/popSepPaper/popR")

getwd()

dir(pattern = "fas", full.names = TRUE)

dir(pattern = "csv", full.names = TRUE)

```

```{r}

dnaAlgn<-read.dna(file="TransAlgn_0718_dicCOI_DsepBlock_noAmbig.fasta", format="fasta")

obj <-DNAbin2genind(dnaAlgn, polyThres = 0.01)

obj

```

obj is a genind object

load in file with pop assignment

```{r}

data.frame <- read.csv("resLocationDic.csv", header = FALSE)

my_strata <- data.frame(region = data.frame$V2, populations = data.frame$V3, sea = data.frame$V4)

strata(obj) <- my_strata

setPop(obj) <- ~populations

#compute pairwise Gst

pairwise_Gst_Nei(obj, linearized = FALSE)

#store as variable

distGst<-pairwise_Gst_Nei(obj, linearized = FALSE)

#write results into file

#write.csv(as.matrix(distGst), file ="GstPopDic_sea.csv")

```

load GEOdist

```{r}

GEOdist <-read.table(file ="GeoDistancesPopDic.csv", sep=",", header=TRUE)

popnames<-as.vector(GEOdist[,1])

GEOdist<-as.matrix(GEOdist[,2:ncol(GEOdist)])

dimnames(GEOdist)<-list(popnames, popnames)

#show matrix

GEOdist

GEOdist2 <-forceSymmetric(GEOdist)

GEOdist3 <-as.dist(GEOdist2)

mantel.randtest(GEOdist3, distGst)

```

and plot results (possibly in console to save pics)

```{r}

plot(GEOdist3, distGst)

abline(lm(distGst ~ GEOdist3))

```

plot something prettier with ggplot, set up a function ggplotRegression()

```{r}

ggplotRegression <- function (fit) {

require(ggplot2)

ggplot(fit$model, aes_string(x = names(fit$model)[2], y = names(fit$model)[1])) +

geom_point() +

geom_smooth(method = "lm", col = "mediumorchid4") +

labs(title = paste("Adj R2=",signif(summary(fit)$adj.r.squared, 5),

" Slope=",signif(fit$coef[[2]], 5),

" P=",signif(summary(fit)$coef[2,4], 5)), x ="marine geographic distance (in km)", y="genetic distance") + theme(plot.title = element_text(color="black", size=12), axis.title.x = element_text(color="black", size=14), axis.title.y = element_text(color="black", size=14))

}

```

prepare data for ggplot, cannot read distance matrix => needs to be in data frame, then plot into file (set png > dev off)

```{r}

GeoDistplot<-melt(as.matrix(GEOdist3))

GenDistplot<-melt(as.matrix(distGst))

GeoxGenplot<-cbind2(GenDistplot,GeoDistplot)

GeoxGenplot2<-GeoxGenplot[,c(1,2,3,6)]

fit1<-lm(GeoxGenplot2$value ~ GeoxGenplot2$value.1)

#png("RplotGeoGenDicPop.png", width = 420, height = 400)

ggplotRegression(fit1)

#dev.off()

```

```{r}

mantelTest<-mantel.randtest(GEOdist3, distGst)

plot(mantelTest)

```

by individuals Sep

```{r}

dnaAlgn<-read.dna(file="TransAlgn_0718_SOcoreBlock.fasta", format="fasta")

AdeGenDist<- dist.dna(dnaAlgn, model="TN93")

write.csv(as.matrix(AdeGenDist), file="Nei_TN93_IndividualSep.csv")

```

```{r}

GEOdistIndividuals <-read.table(file="GEOdistances_individualsSep.csv", sep=",", header=TRUE)

GEOdistIndividuals<-as.matrix(GEOdistIndividuals[,2:ncol(GEOdistIndividuals)])

GEOdistIndividuals2 <-forceSymmetric(GEOdistIndividuals)

GEOdistIndividuals3 <-as.dist(GEOdistIndividuals2)

```

```{r}

plot(GEOdistIndividuals3, AdeGenDist)

abline(lm(AdeGenDist ~ GEOdistIndividuals3))

```

dic by individuals

load in data and create genetic distance matrix

```{r}

dnaAlgn<-read.dna(file="TransAlgn_0718_dicCOI_DsepBlock_noAmbig.fasta", format="fasta")

AdeGenDist<- dist.dna(dnaAlgn, model="TN93")

#write.csv(as.matrix(AdeGenDist), file="Nei_TN93_IndividualSep.csv")

```

load in geographic distance matrix

```{r}

GEOdistIndividuals <-read.table(file="GeoDistancesDicIndividuals.csv", sep=",", header=TRUE)

GEOdistIndividuals<-as.matrix(GEOdistIndividuals[,2:ncol(GEOdistIndividuals)])

GEOdistIndividuals2 <-forceSymmetric(GEOdistIndividuals)

GEOdistIndividuals3 <-as.dist(GEOdistIndividuals2)

```

#here could be mantel test

prepare data for plotting in ggplot, + plot + save to png file

```{r}

GeoDistplot<-melt(as.matrix(GEOdistIndividuals3))

GenDistplot<-melt(as.matrix(AdeGenDist))

GeoxGenplot<-cbind2(GenDistplot,GeoDistplot)

GeoxGenplot2<-GeoxGenplot[,c(1,2,3,6)]

fit1<-lm(GeoxGenplot2$value ~ GeoxGenplot2$value.1)

png("RplotGeoGenDicInd.png", width = 420, height = 400)

ggplotRegression(fit1)

dev.off()

```

replot Sep pops

```{r}

dnaAlgn<-read.dna(file="TransAlgn_0718_SOcoreBlock.fas", format="fasta")

obj <-DNAbin2genind(dnaAlgn, polyThres = 0.01)

obj

```

```{r}

data.frame <- read.csv("resLocationSep.csv", header = FALSE)

my_strata <- data.frame(region = data.frame$V2, populations = data.frame$V3, sea = data.frame$V4)

strata(obj) <- my_strata

setPop(obj) <- ~populations

#compute pairwise Gst

pairwise_Gst_Nei(obj, linearized = FALSE)

#store as variable

distGst<-pairwise_Gst_Nei(obj, linearized = FALSE)

```

```{r}

GEOdist <-read.table(file ="popSepSortedGeoDistances.csv", sep=",", header=TRUE)

popnames<-as.vector(GEOdist[,1])

GEOdist<-as.matrix(GEOdist[,2:ncol(GEOdist)])

dimnames(GEOdist)<-list(popnames, popnames)

#show matrix

GEOdist

GEOdist2 <-forceSymmetric(GEOdist)

GEOdist3 <-as.dist(GEOdist2)

```

```{r}

GeoDistplot<-melt(as.matrix(GEOdist3))

GenDistplot<-melt(as.matrix(distGst))

GeoxGenplot<-cbind2(GenDistplot,GeoDistplot)

GeoxGenplot2<-GeoxGenplot[,c(1,2,3,6)]

fit1<-lm(GeoxGenplot2$value ~ GeoxGenplot2$value.1)

png("RplotGeoGenSepPop.png", width = 420, height = 400)

ggplotRegression(fit1)

dev.off()

```

**regressions**

```{r}

da = read.csv("Da_Dic_Sep.csv", header = TRUE)

da<-na.omit(da)

head(da)

host_da <- da[ ,1]

parasite_da <- da[ ,2]

```

```{r}

nuc = read.csv("av_nuc_diff.csv", header = TRUE)

nuc<-na.omit(nuc)

head(nuc)

host_nuc <- nuc[ ,1]

parasite_nuc <- nuc[ ,2]

```{r}

reg_da <- lm(parasite_da ~ host_da)

png("Rplot_Da.png", width = 600, height = 600)

ggplotRegression(reg_da)

dev.off()

svg("Rplot_Da.svg")

ggplotRegression(reg_da)

dev.off()

```

**AMOVA**

dnaAlgn<-read.dna(file="TransAlgn_0718_dicCOI_DsepBlock_noAmbig.fasta", format="fasta")

obj <-DNAbin2genind(dnaAlgn, polyThres = 0.01)

obj

#load in columns with pop assignment, can be more then one

data.frame <- read.csv("resLocationDic.csv", header = FALSE)

#set pop structure as strata

my_strata <- data.frame(region = data.frame$V2, populations = data.frame$V3, sea = data.frame$V4)

strata(obj) <- my_strata

setPop(obj) <- ~populations

poppr.amova(obj, hier = ~region)

poppr.amova(obj, hier = ~populations)

dnaAlgn<-read.dna(file="v2_TransAlgn_0718_SOcoreBlock.fasta", format="fasta")

obj <-DNAbin2genind(dnaAlgn, polyThres = 0.01)

obj

#load in columns with pop assignment, can be more then one

data.frame <- read.csv("v2_resLocationSep.csv", header = FALSE)

#set pop structure as strata

my_strata <- data.frame(region = data.frame$V2, populations = data.frame$V3, sea = data.frame$V4)

strata(obj) <- my_strata

setPop(obj) <- ~populations

poppr.amova(obj, hier = ~populations)

**permutation test for AMOVA**

set.seed(1999)

Amova <- poppr.amova(obj, hier = ~populations)

AmovaSignif   <- randtest(Amova, nrepet = 999)

plot(AmovaSignif)

AmovaSignif

**PCoA**

Script used for compuation (mainly inspired by <https://github.com/wilkox/doPCoA/blob/master/R/do_PCoA.R> and Alenka Bartonova)

---

title: "R Notebook PCOA"

output: html_notebook

---

libraries

```{r}

library(ape)

library("reshape2")

library("vegan")

library("dplyr")

```

assignement of samples into pops (not necessary)

genetic distances

```{r}

dnaAlgn<-read.dna(file="TransAlgn_0718_SOcoreBlock.fasta", format="fasta")

AdeGenDist<- dist.dna(dnaAlgn, model="TN93")

```

pcoa

```{r}

do_PCoA <- function(DistanceMatrix) {

 # Return error if distance matrix is not a distance matrix

 if (class(DistanceMatrix) != "dist") {

stop("Distance matrix must be an object of type dist. See ?read_dist for a

     way to read a distance matrix from a file into a dist object.")

 }

 # Run the PCoA

 PCoA <- cmdscale(DistanceMatrix, k = 2, eig = TRUE)

 # Extract PCoA coordinates

 Coordinates <- data.frame(

Sample = row.names(PCoA$points),

PCoA1 = PCoA$points[,1],

PCoA2 = PCoA$points[,2],

row.names = NULL

 ) %>%

as.tbl()

 # Calculate variance explained

 # Method is from http://r-sig-ecology.471788.n2.nabble.com/\

 # Variability-explanations-for-the-PCO-axes-as-in-Anderson-and-Willis-2003-\

 # td6429547.html

 Eigenvalues <- eigenvals(PCoA)

 Variance <- Eigenvalues / sum(Eigenvalues)

 Variance1 <- 100 * signif(Variance[1], 2)

 Variance2 <- 100 * signif(Variance[2], 2)

 # Return

 Result <- list(

Coordinates = Coordinates,

Variance1 = Variance1,

Variance2 = Variance2

 )

 return(Result)

}

```

```{r}

do_PCoA(AdeGenDist)

```

plot check

```{r}

PCoA <- cmdscale(AdeGenDist, k = 2, eig = TRUE)

plot(PCoA$points[,1:2],pch=21,col="black", cex=1.3)

```

Color assignment to Groups

```{r}

groups <- read.csv("v2_resLocationSepPcoa.csv", header = FALSE)

colors()[41:60] #muzu menit cisla, ukazuje mi nazvy moznych barev

palette("default") #nastavuje paletu zpet na defaultni barvy

cc <- palette()

palette(c(cc,"purple","brown","pink","coral1", "blue4", "chocolate1", "blueviolet")) #pripojim dalsi barvy k palete

palette()

plot(PCoA$points[,1:2],bg=groups$V2, pch=21,col="black", cex=1.3)

legend(-0.0045,0.0027,legend = unique(groups$V2),fill = unique(groups$V2))

```

#colors then changed in Inkscape
